# Site‐Selective, Multistep Functionalizations of CO_2_‐Based Hyperbranched Poly(alkynoate)s toward Functional Polymetric Materials

**DOI:** 10.1002/advs.202000465

**Published:** 2020-07-08

**Authors:** Bo Song, Rongyuan Zhang, Rong Hu, Xu Chen, Dongming Liu, Jiali Guo, Xiaotian Xu, Anjun Qin, Ben Zhong Tang

**Affiliations:** ^1^ State Key Laboratory of Luminescent Materials and Devices Guangdong Provincial Key Laboratory of Luminescence from Molecular Aggregates Center for Aggregation‐Induced Emission South China University of Technology Guangzhou 510640 China; ^2^ Department of Urology The First Affiliated Hospital of Soochow University 188 Shizi RD Suzhou 215006 China; ^3^ Department of Chemistry Hong Kong Branch of Chinese National Engineering Research Centre for Tissue Restoration and Reconstruction Institute for Advanced Study and Department of Chemical and Biological Engineering The Hong Kong University of Science & Technology Clear Water Bay Kowloon Hong Kong China

**Keywords:** aggregation‐induced emission, carbon dioxide fixation, functionalization, hyperbranched polymers, site‐selective multistep functionalization

## Abstract

Hyperbranched polymers constructed from CO_2_ possess unique architectures and properties; however, they are difficult to prepare. In this work, CO_2_‐based, hyperbranched poly(alkynoate)s (hb‐PAs) with high molecular weights and degrees of branching are facilely prepared under atmospheric pressure in only 3 h. Because hb‐PAs possess two types of ethynyl groups with different reactivities, they can undergo site‐selective, three‐step functionalizations with nearly 100% conversion in each step. Taking advantage of this unique feature, functional hb‐PAs with versatile properties are constructed that could be selectively tailored to contain hydrophilic oligo(ethylene glycol) chains in their branched chains, on their periphery, or both via tandem polymerizations. Hyperbranched polyprodrug amphiphiles with high drug loading content (44.3 wt%) are also generated, along with an artificial light‐harvesting system with high energy transfer efficiency (up to 92%) and white‐light‐emitting polymers. This work not only provides an efficient pathway to convert CO_2_ into hyperbranched polymers, but also offers an effective platform for site‐selective multistep functionalizations toward functional polymeric materials.

## Graphical abstract

Hyperbranched poly(alkynoate)s are facilely prepared from CO_2_, serving as a versatile platform for site‐selective multistep functionalizations, based on which polyprodrug amphiphiles with high drug loading content, artificial light‐harvesting system with high energy transfer efficiency and white light‐emitting system are constructed.

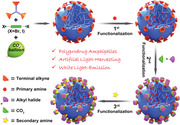

## 1 Introduction

As an abundant, inexpensive, nontoxic, and renewable C1 resource, carbon dioxide (CO_2_) has been converted to linear polymers such as polycarbonates,^[^ ^1^, ^2^, ^3^, ^4^, ^5^, ^6^, ^7^, ^8^, ^9^ ^]^ poly(urethane)s,^[^ ^10^ ^]^ polyureas,^[^ ^11^ ^]^ and poly(urethane‐carbonate)s.^[^ ^12^ ^]^ However, due to the limited flexibility in designing multi‐functionalized monomers, studies using CO_2_ to construct polymers with topological structures, such as hyperbranched polymers (HBPs), have rarely been reported.^[^ ^13^ ^]^ Developing CO_2_‐based HBPs is of great significance because this process can not only increase the incorporation content of CO_2_ into polymers, but also produce polymers with advanced architectures and unique properties. Thus, a facile and versatile polymerization strategy for the synthesis of CO_2_‐based HBPs is highly desirable.

**Figure 1:**
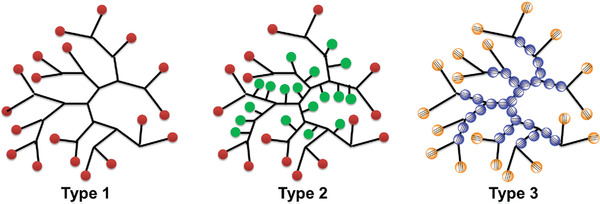
Different types of HBPs for one‐ or multistep functionalizations (balls in the structures mean functional groups, different colours mean different functional groups or same functional groups with differentiated reactivity).

The functionalization of HBPs is vital for modulating their architectures and properties for diverse applications in the fields of drug delivery, light harvesting, catalysis matrices, etc.^[^ ^14^ ^]^ Nowadays, due to the large number of readily accessible terminal functional groups of most HBPs, tremendous efforts have been made toward developing one‐step functionalizations of HBPs (**Figure** **1**, Type 1).^[^ ^15^ ^]^ Functionalizations with more than one step have only been realized for a few elaborately design HBPs (Figure 1, Type 2). For example, Haag et al. reported a hyperbranched polyglycerol whose terminal glycerol units were selectively converted into acetals/ketals while the linear units were functionalized with alkyl halides.^[^ ^16^, ^17^ ^]^ Gao et al. reported poly(tertiary amino methacrylate)s that could be functionalized via the Menschutkin reaction and Cu(I)‐catalyzed azide‐alkyne cycloaddition.^[^ ^18^ ^]^ Khan et al. also constructed a type of HBP containing two reactive hydroxyl and epoxide groups for two‐step functionalizations.^[^ ^19^ ^]^ Among these reports, the branched chains of HBPs remained intact because they were composed of saturated C—C bonds that could not be further functionalized. In addition, some functionalization reactions for HBPs are not efficient enough, which might influence their architectures and properties.

The polymerization of CO_2_ with alkyne and alkyl dihalide monomers could readily produce poly(alkynoate)s with ester activated ethynyl groups that could efficiently react with various functional groups such as azides, amino groups, thiols, and hydroxyl groups.^[^ ^20^, ^21^ ^]^ Therefore, we envisaged that the CO_2_‐based hyperbranched poly(alkynoate)s (hb‐PAs, Figure 1, Type 3) could overcome the aforementioned limitations and be used as an effective platform for site‐selective, multistep functionalizations with quantitative conversions that modulate their branched chains and peripheries to construct multifunctional materials.

**Figure 2:**
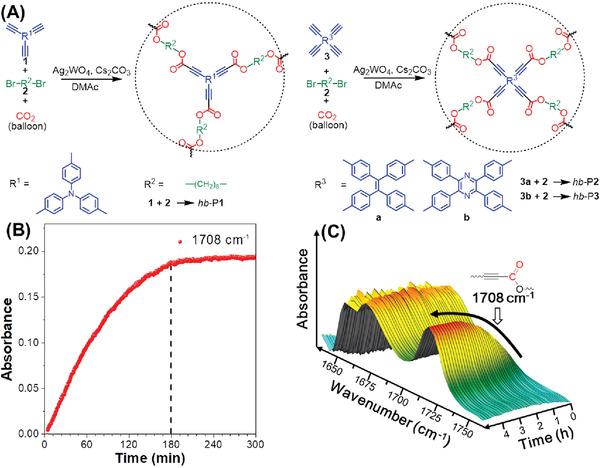
A) Synthetic routes to hb‐PAs via three‐component polymerizations of the monomers CO_2_, 1 (or 3) and 2. B) Kinetic profile and C) 3D FT‐IR profile of the polymerization.

In this work, CO_2_‐based hb‐PAs were rationally designed and easily prepared via the following monomer strategy (**Figure** **2**A): tri/tetrayne (A*_n_*) + alkyl dihalide (B_2_) + CO_2_ (C).^[^ ^22^ ^]^ The resultant hb‐PAs possessed two types of ethynyl groups in their branched chains and on their peripheries, which showed different reactivities (Figure 1, Type 3). As a result, they could undergo site‐selective, three‐step functionalizations with nearly 100% conversion in each step. Furthermore, via a simple, one‐pot, two/three‐step, four/five‐component tandem polymerization, hb‐PAs could be selectively tailored to have hydrophilic oligo(ethylene glycol) (OEG) chains in their branched chains, their peripheries, or both. Taking advantage of this simple and efficient site‐selective functionalization strategy, we obtained various advanced functional materials. For example, hyperbranched polyprodrug amphiphiles were generated with high drug (doxorubicin, DOX) loading content (44.3 wt%), excellent, sustained acid‐targeted release, and higher in vitro anti‐cancer efficacy than that of free DOX. Moreover, an artificial light‐harvesting system with high energy transfer efficiency (up to 92%) and white light‐emitting polymeric materials were also constructed.

## 2 Results and Discussion

### 2.1 Synthesis of Hyperbranched Poly(alkynoate)s

In general, gelation could be a problem when preparing the hb‐PAs via the “A*_n_* + B_2_ + C” monomer strategy. To obtain soluble hb‐PAs, the polymerization should be quenched before the gelation point.^[^ ^23^ ^]^ From the textbook of polymer chemistry, it is known that the gelation point can be calculated from the Flory Statistics.^[^ ^24^ ^]^ For an A_2_ + B_2_ + A*_n_* (*n* > 2) monomer system, the Flory statistics can be simplified into Equation (1)

(1) $$\begin{array}{c} {\left(p_{A}\right)}_{C}=\;\frac{1}{{\left[r+r\rho (n-2)\right]}^{1/2}} \end{array}$$

where (*p*_A_)_*C*_ is the critical reaction extent of group A, *r* is the molar ratio of groups B and A, and *ρ* is the percentage of group A from monomer A*_n_*. Specifically, in our “A*_n_* + B_2_ + C” co‐monomer system, *ρ* = 1, so Equation (1) can be further simplified into Equation (2)

(2) $$\begin{array}{c} {\left(p_{A}\right)}_{C}=\;\frac{1}{{\left[(n-1)r\right]}^{1/2}} \end{array}$$

Therefore, when equal concentrations of A_3_ or A_4_ and B_2_ monomers were used, the gelation points (*p*_A_)_*C*_ were calculated to be 0.866 and 0.816 for “A_3_ + B_2_” and “A_4_ + B_2_” co‐monomer system, respectively. In these cases, group B was consumed completely and the maximal reaction extents of the group A in “A_3_ + B_2_” and “A_4_ + B_2_” co‐monomer system were determined to be 0.667 and 0.50, respectively, which are far lower than the (*p*_A_)_*C*_ values. Thus, gelation might not occur when using equal molar ratios of A_3_ or A_4_ and B_2_ monomers.

Based on the above theoretical calculation, we first tried to polymerize CO_2_ with an equal molar ratio of tris(4‐ethynylphenyl)amine (1, A_3_) and 1,8‐dibromooctane (2, B_2_) in the presence of the catalytic system Ag_2_WO_4_/Cs_2_CO_3_ in *N*,*N*‐dimethylacetamide (DMAc) at 80 °C under atmospheric pressure (Figure 2A and Table S1, Supporting Information). After 3 h, a soluble product was obtained in 95% yield, as expected. However, when we changed the molar ratio of 1 and 2 to 2:3 (equal molar ratio of reactive groups), the gelation easily occurred and only insoluble product was obtained after 3 h. Therefore, an equal molar ratio in relation to the monomers instead of reactive groups was adopted to construct soluble hb‐PAs in this work.

A time course of the polymerization was then followed (Table S2, Supporting Information). The weight‐average molecular weight (*M*_w_) values of the products increased within 3 h. Extending the reaction time to 4 h slightly improved both the yields and *M*_w_ values of the products. This could be explained by the reaction mechanism shown in Scheme S1 (Supporting Information).^[^ ^22^ ^]^ First, the Ag^+^ catalytic species coordinates with the ethynyl group of triyne 1 to form intermediate A. Then, the silver acetylide B is generated from A with the aid of Cs_2_CO_3_. Subsequent insertion of C, a CO_2_―WO_4_^2−^ adduct, into B readily forms silver phenylpropiolate D with the release of WO_4_^2−^. Finally, D reacts with alkyl dihalide 2 to produce the hb‐PA via esterification. When the polymerization was performed for 3 h, the alkyl dihalide might have been completely consumed due to the equivalent molar ratio of the monomers. To better understand the kinetic profile of the polymerization, we used in situ Fourier‐transform infrared (FT‐IR) spectroscopy to monitor the reaction process. As shown in Figure 2B,C, a new band attributed to the C=O stretching vibration formed at 1708 cm^−1^, which increased in intensity over 3 h but remained almost unchanged afterward. This confirmed that the polymerization is complete after at least 3 h; thus, we used 3 h as our optimal polymerization time.

Applying these optimized conditions, we polymerized equal molar ratios of tetraynes 3a and 3b (Scheme S2, Supporting Information), 2, and CO_2_ in an “A_4_ + B_2_ + C” monomer strategy to synthesize the corresponding hb‐PAs (Figure 2A). As shown in Table S3 (Supporting Information), the polymerizations using either “A_3_ + B_2_ + C” or “A_4_ + B_2_ + C” monomer strategies could furnish soluble hb‐PAs with *M*_w_ values of up to 21 400 and absolute values of up to 226 700 (Figures S1–S3, Supporting Information), demonstrating that this polymerization strategy is a simple and successful way to generate hb‐PAs from CO_2_.

The resultant hb‐PAs showed good solubility in commonly used organic solvents, such as tetrahydrofuran (THF), dichloromethane (DCM), *N,N*‐dimethylformamide (DMF), and chloroform. These polymers are also thermally stable. As shown in Figure S4 (Supporting Information), the temperatures that incurred 5% weight loss (*T*_d_), determined by thermogravimetric analysis (TGA), are in the range of 374 to 395 °C under nitrogen. All the polymers retained 60–80% of their original weights after being heated to 800 °C, giving them heat‐resistant properties for potential use in various applications.

### 2.2 Structural Characterization

The solubility of the resultant polymers allowed us to characterize their structures by FT‐IR spectroscopy and ^1^H and ^13^C nuclear magnetic resonance (NMR) spectroscopies, and satisfactory structural analysis data were obtained. To facilitate the structural characterization, the Ag_2_WO_4_/Cs_2_CO_3_‐catalyzed reaction of triyne 1, 1‐bromooctane (4), and CO_2_ in DMAc at 80 °C under atmospheric pressure for 3 h was carried out to prepare model compounds 5, 6, and 7 (Scheme S3, Supporting Information). Fortunately, 5, 6, and 7 could be easily separated and purified by silica gel column chromatography.

The FT‐IR, ^1^H NMR, and ^13^C NMR spectra of polymer hb‐P1, model compounds 5, 6, and 7, and their corresponding monomer (1) were analyzed. In the FT‐IR spectra (Figure S5, Supporting Information), new C=O stretching vibration bands are observed at 1708 cm^−1^ for 5, 6, 7, and hb‐P1, confirming successful polymerization. The C≡C stretching vibrations at 2208 and 2101 cm^−1^ and the ≡C—H stretching vibration at 3287 cm^−1^ were observed in the spectra of hb‐P1, 5 and 6, indicating that there are two types of ethynyl groups in the branched chains and on the peripheries of hb‐P1.

The ^1^H and ^13^C NMR spectra provided more detailed information about the polymer structures. From the ^1^H NMR spectra (Figure S6, Supporting Information), we found that the ethynyl protons of 1 resonated at *δ* 3.11 ppm and remained in the spectra of 5, 6, and hb‐P1. Meanwhile, new resonances emerged at *δ* 4.19 ppm in the spectra of 5, 6, 7, and hb‐P1, representing the protons of the methylene group adjacent to the generated ester groups. In the ^13^C NMR spectra of 5, 6, 7, and hb‐P1 (Figure S7, Supporting Information), the resonances of the ester carbons and methylene carbons adjacent to the generated ester groups appeared at *δ* 154.37−154.50 ppm and 66.45−66.62 ppm, respectively. Additionally, two types of C≡C resonant peaks at *δ* 86.59−77.51 ppm were also observed in the spectra of hb‐P1, 5, and 6. These results are in good agreement with the FT‐IR and ^1^H NMR spectral analysis, further confirming the polymer structures. Similar results were also observed in the FT‐IR and ^1^H and ^13^C NMR spectra of hb‐P2 and hb‐P3 (Figures S8–S13, Supporting Information).

An important structural parameter for an HBP is its degree of branching (DB), which is often determined by ^1^H NMR spectral analysis.^[^ ^14^, ^25^, ^26^ ^]^ As shown in Figure S14A (Supporting Information), there are three structural components in hb‐P1: dendritic (*D*), linear (*L*), and terminal (*T*) units. By comparing the ^1^H NMR spectrum of hb‐P1 with those of its monomer (1) and model compounds 5, 6, and 7 (Figure S14B–F, Supporting Information), we can conclude that two ethynyl proton resonances of hb‐P1 correspond to the *T* and *L* units. Thus, the DB value of hb‐P1 was deduced to be 0.61 (see the Supporting Information for details), which is higher than those of “conventional” HBPs (commonly ≈0.5) and further confirms the hyperbranched structures of these polymers.

### 2.3 Aggregation‐Induced Emission

Thanks to the functional tolerance of this polymerization, tetraphenylethene (TPE) and tetraphenylpyrazine (TPP) units featuring aggregation‐induced emission (AIE) characteristics can be easily introduced into the skeletons of hb‐PAs. Therefore, we systematically investigated the emission behaviors of hb‐P2 and hb‐P3 which contain TPE and TPP units, respectively, in THF/water mixtures with different water fractions (*f*_w_, Figures S15 and S16, Supporting Information) and determined their absolute fluorescence quantum yields (*Φ*_F_, Table S4, Supporting Information). We found that both of them are AIE‐active. They emit faintly in THF, but adding a poor solvent like water increased the emission intensity. The highest emission values were recorded in the THF/water mixtures with *f*_w_ values of 90% and 80% for hb‐P2 and hb‐P3, respectively, due to the restriction of intramolecular motion.^[^ ^27^ ^]^ The *Φ*_F_ of hb‐P2 in its film state is 49%, which is much higher than that of most AIE‐active HBPs and likely because the flexible branched chains allow the AIE units to rotate freely in solution and to pack more tightly in the aggregate state.^[^ ^28^ ^]^ As shown in Figure S17 (Supporting Information), the polymer aggregates are almost spherical with diameters of ≈100 nm, which might be due to their hyperbranched structures.

### 2.4 Site‐Selective Three‐Step Functionalizations

From the structural characterization, we could conclude that our resultant hyperbranched poly(alkynoate)s maintain highly reactive alkynoate and terminal ethynyl groups in their branched chains and on their peripheries, respectively. The alkynoate groups can selectively convert into aminoacrylate groups by a catalyst‐free amino‐yne click reaction, while the terminal ethynyl groups remain intact.^[^ ^29^ ^]^ Moreover, the terminal ethynyl groups on the peripheries of hb‐PAs can be easily converted to reactive alkynoate groups by the post‐reaction of CO_2_ and alkyl halides under the same reaction conditions as the polymerizations. Taking advantage of these transformations, site‐selective, three‐step functionalizations of hb‐PAs were performed.

**Figure 3:**
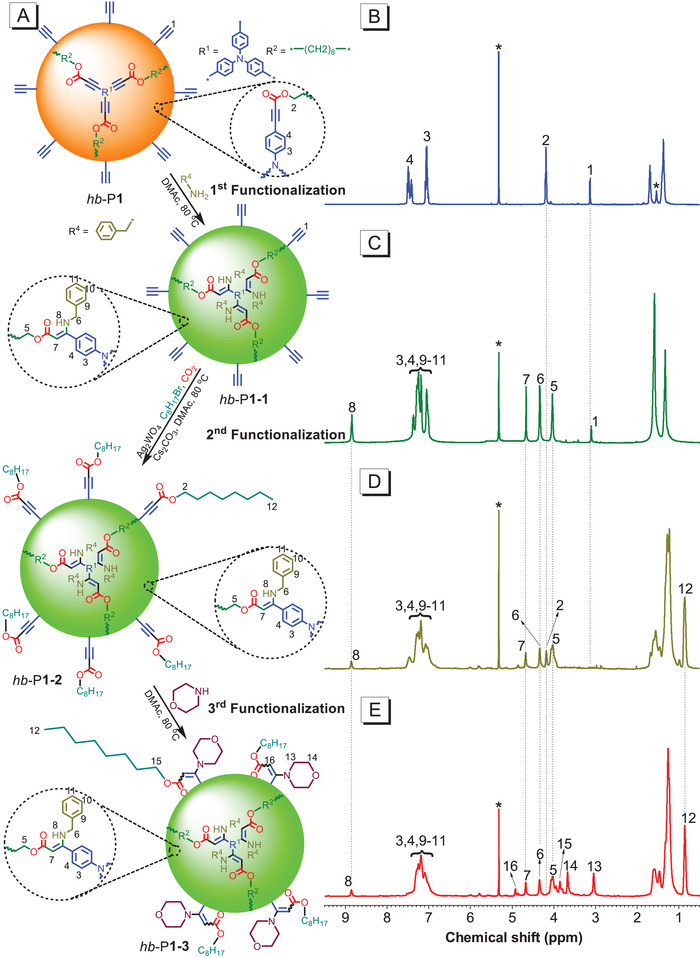
Site‐selective multi‐step functionalizations of A) hb‐P1 and B) ^1^H NMR spectra of polymer hb‐P1, C) hb‐P1‐1, D) hb‐P1‐2 and E) hb‐P1‐3 in DCM‐*d*_2_. The solvent peaks are marked with asterisks.

Herein, hb‐P1 was used as a model to demonstrate these site‐selective, three‐step functionalizations (**Figure** **3**A). First, the primary amine of benzylamine was reacted with hb‐P1 in DMAc at 80 °C in air without adding any catalyst, and the modified product, hb‐P1‐1, was obtained after 5 h. As shown in Figure 3B,C, the resonance at *δ* 4.19 ppm in the ^1^H NMR spectrum of hb‐P1 was absent in that of hb‐P1‐1, indicating that the alkynoate groups in the branched chains of the former have been fully consumed because of the high reactivity of amino groups with activated ethynyls. Furthermore, amino groups do not react with terminal ethynyl groups under these conditions, so the resonance at *δ* 3.10 ppm representing these ethynyl protons was also observed in the ^1^H NMR spectrum of hb‐P1‐1. The integrals of the resonant peaks at *δ* 3.10 ppm in the spectra of hb‐P1 and hb‐P1‐1 were calculated and compared to those of the protons of the methylene groups adjacent to the ester groups (Figure S18, Supporting Information). It was again confirmed that 100% of the terminal ethynyl groups remained, clearly showing site‐selective functionalization.

Second, 1‐bromooctane (4, Scheme S3, Supporting Information) and CO_2_ (via a balloon) were introduced and reacted with the terminal ethynyl groups of hb‐P1‐1 in the presence of Ag_2_WO_4_/Cs_2_CO_3_ in DMAc at 80 °C, and hb‐P1‐2 was generated after 12 h. From the ^1^H NMR spectrum of hb‐P1‐2 in Figure 3D, we can see that the resonance at *δ* 3.10 ppm disappeared and a new peak at *δ* 4.18 ppm appeared, indicating that the terminal ethynyl groups on the periphery of hb‐P1‐1 were fully converted to alkynoate groups. The other resonant peaks in the hb‐P1‐2 spectrum are inherited from that of hb‐P1‐1, indicating that the aminoacrylate groups remain intact during this functionalization.

Third, the secondary amine of morpholine was allowed to react with the newly generated alkynoate groups on the periphery of hb‐P1‐2 in DMAc at 80 °C in air, and hb‐P1‐3 was produced after 12 h. As shown in Figure 3E, the resonant peak at *δ* 4.18 ppm in the ^1^H NMR spectrum of hb‐P1‐2 was absent in that of hb‐P1‐3, suggesting that the alkynoate groups on the periphery of hb‐P1‐2 were fully consumed. The remaining resonances in the ^1^H NMR spectrum of hb‐P1‐3 (Figure 3C–E) reflect those in the spectrum of hb‐P1‐2, indicating that the added reagent only reacted with the target functional group. This was true for each step, where nearly 100% conversion was achieved. Therefore, structure‐ and sequence‐controlled poly(aminoacrylate)s were successfully obtained via the site‐selective three‐step functionalizations of hb‐PAs. Moreover, this technique is an effective tool for producing functional polymeric materials with unique structures and versatile properties that could be used for performing site‐selective tandem polymerizations, constructing hyperbranched polyprodrug amphiphiles, artificial light‐harvesting, and producing white‐light emission polymeric materials.

### 2.5 Site‐Selective Tandem Polymerization

Because the three‐component polymerization and the amino‐yne click reaction could be carried out under the same conditions, we combined the synthesis and site‐selective multi‐step functionalization of hb‐PAs into a one‐pot, tandem polymerization to generate amphiphilic hyperbranched polymers. Compared with traditional “step‐by‐step” operations, the tandem polymerization can combine multiple steps into one synthetic operation, which could save time and simplify experimental procedures so that structurally complex polymers can be prepared from simple and readily available monomers.^[^ ^30^ ^]^ Herein, the hydrophilic bromo‐ and amino‐terminated OEG derivatives 11 and 12, respectively, shown in Figure S19A (Supporting Information), were chosen to demonstrate the site‐selective tandem polymerizations.

We first polymerized CO_2_, 1, and 2 in the presence of Ag_2_WO_4_/Cs_2_CO_3_. Then, without any isolation and purification, the OEG derivatives were added for the tandem polymerizations to selectively introduce OEG chains to the specific sites of the polymers via three paths (Figure S19, Supporting Information). When only 11 was added, the OEG chains were selectively functionalized on the peripheries of hb‐PAs to furnish hb‐P1‐4 (Figure S19A (Supporting Information), Path A); whereas, when only 12 was introduced through the amino‐yne click reaction, the OEG chains could be selectively functionalized in the branched chains of hb‐PAs to yield hb‐P1‐5 (Figure S19A (Supporting Information), Path B). When 11 was added first and then followed by 12, the OEG chains could be functionalized both in the branched chains and on the peripheries of the polymer via the Ag_2_WO_4_‐catalyzed, three‐component reaction and the amino‐yne click reaction, respectively, to generate hb‐P1‐6 (Figure S19A (Supporting Information), Path C). Moreover, the ^1^H NMR spectral analysis indicates that the target ethynyl groups were fully consumed during the one‐pot, two/three‐step, four/five‐component tandem polymerizations, and the resultant amphiphilic polymers, hb‐P1‐4, hb‐P1‐5, and hb‐P1‐6, have well‐defined structures (Figure S19B–D, Supporting Information). This site‐selective tandem polymerization strategy is effective for quickly constructing complex architectures of HBPs via simple synthetic operations.

### 2.6 Hyperbranched Polyprodrug Amphiphiles

Our resultant hb‐poly(aminoacrylate)s can be formed from hb‐PAs via either a site‐selective three‐step functionalization or a tandem reaction. They contain bulky enamine groups that are reportedly acid sensitive and could be converted to amino and aldehyde/ketone groups under weak acidic conditions,^[^ ^31^, ^32^ ^]^ which could facilitate them to be used as stimuli‐responsive polyprodrug amphiphiles.^[^ ^33^, ^34^, ^35^, ^36^ ^]^

**Figure 4:**
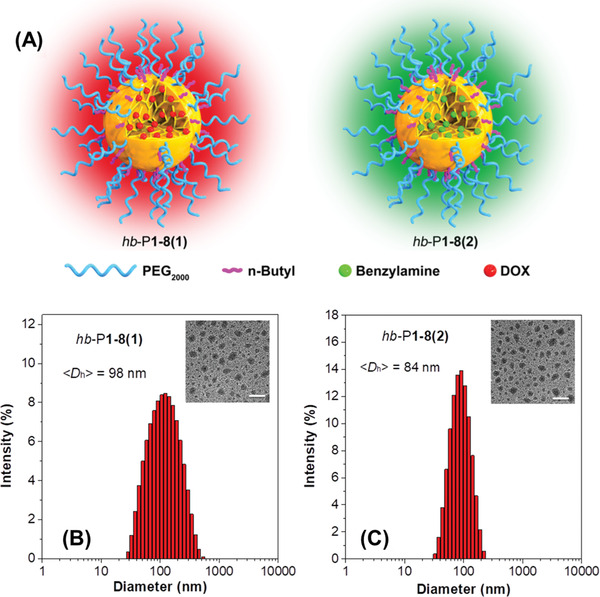
A) Schematic illustration of hyperbranched polyprodrug amphiphiles and B,C) Hydrodynamic diameter distribution of hb‐P1‐8(1) and hb‐P1‐8(2) in water (Inset: TEM image determined for the aqueous dispersion of hb‐P1‐8(1) and hb‐P1‐8(2)). Scale bar = 200 nm.

To test the potential application of our hb‐PAs as acid‐triggered hyperbranched polyprodrug amphiphiles for controlled drug release (**Figure** **4**A), we synthesized an amphiphilic polymer hb‐P1‐7 from CO_2_, 1, 2, hydrophobic 1‐butyl iodide, and hydrophilic, bromo‐terminated poly(ethylene glycol) (mPEG‐Br) via a one‐pot, three‐step, five‐component tandem polymerization (Scheme S4 and Figure S20, Supporting Information). Then, DOX, which contains aliphatic amino groups, was chosen as a model anti‐cancer drug to undergo the amino‐yne click reaction with hb‐P1‐7 and polyprodrug amphiphile hb‐P1‐8(1) was readily generated. For comparison, benzylamine was also reacted with hb‐P1‐7 to produce hb‐P1‐8(2) under the same conditions. Their structures were unambiguously characterized by ^1^H NMR spectra (Figures S21 and S22, Supporting Information). Thanks to their amphiphilic properties, both hb‐P1‐8(1) and hb‐P1‐8(2) self‐assemble in aqueous solutions and furnish nanoparticles (NPs) with diameters of 98 and 84 nm, respectively, which were determined by dynamic laser scattering (DLS) analysis (Figure 4B,C). The hb‐P1‐8(1) NPs possessed high drug loading content (44.3 wt%), which was analyzed by UV–vis spectrophotometry (Figure S23, Supporting Information).

Next, we used ^1^H NMR spectroscopy to study the acid‐triggered release of benzylamine from the hb‐P1‐8(2) (Figure S24A, Supporting Information). Due to the core‐shell structure, the NPs of hb‐P1‐8(2) in D_2_O only showed the PEG proton resonances; whereas, the benzylamine peaks appeared when the NPs were in a D_2_O/D_2_SO_4_ solution (pH = 5) and an increase in peak intensity was observed with the extension of time (Figure S24B, Supporting Information).

**Figure 5:**
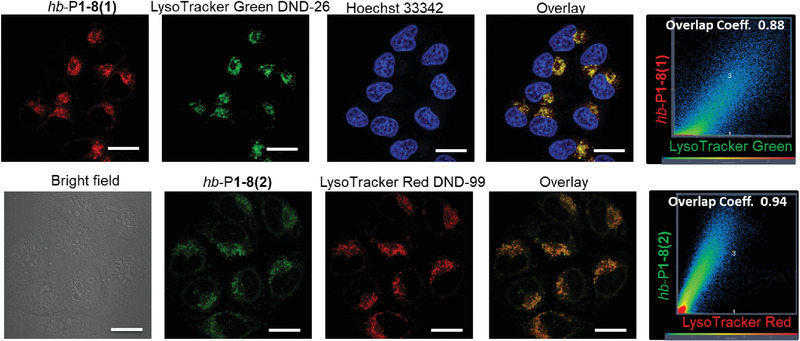
CLSM images of HeLa cells incubated with hb‐P1‐8(1) or hb‐P1‐8(2) NPs after incubation for 3 h, followed with co‐stained by Lysotracker and Hoechst 33 342. [HBPs] = 5 µg mL^−1^, [Lysotracker] = 200 × 10^−9^ m, [Hoechst 33 342] = 1 × 10^−6^ m. *λ*_ex_: 514 nm for hb‐P1‐8(1), 488 nm for LTG, 405 nm for hb‐P1‐8(2) and Hoechst 33 342, 543 nm for LTR. Scale bar = 20 µm.

We then investigated the in vitro release of DOX from hb‐P1‐8(1) NPs in phosphate buffered saline (PBS) at different pH levels. As shown in Figure S25 (Supporting Information), accumulative release of DOX could reach ≈78% at a pH value of 5.0 after dialysis for 150 h, which was used to mimic lysosomal environment of the tumor cells. Meanwhile, the NPs in neutral PBS (pH 7.4) showed ≈16% accumulative drug release, which might be attributed to the dynamic equilibrium of the enamine groups. Afterward, the in vitro cellular uptake and imaging of hb‐P1‐8(1) and hb‐P1‐8(2) NPs were studied using confocal laser scanning microscopy (CLSM). Remarkable red and green fluorescence were observed in HeLa cells after incubation with 5 µg mL^−1^ of hb‐P1‐8(1) or hb‐P1‐8(2) NPs for 3 h (**Figure** **5**). Co‐staining experiments with commercial lysosome (LysoTracker Green DND‐26 (LTG) and LysoTracker Red DND‐99 (LTR)) and nucleus (Hoechst 33 342) imaging agents showed that the cell staining regions of hb‐P1‐8(1) NPs with LTG and hb‐P1‐8(2) NPs with LTR overlapped very well (Figure 5), and their Pearson's correlation coefficients, commonly used to quantify the overlapping extent,^[^ ^37^ ^]^ were calculated to be 0.88 and 0.94, respectively. The good specificity of hb‐P1‐8(1) and hb‐P1‐8(2) NPs toward lysosomes might be ascribed to their cellular uptake mechanism of the endocytosis pathway. After confirming the effective cellular uptake mechanisms of both hb‐P1‐8(1) and hb‐P1‐8(2) NPs, their in vitro cytotoxicity was then investigated. Although hb‐P1‐8(2) NPs were almost nontoxic to HeLa cells at concentrations up to 100 µg mL^−1^, because no drug was incorporated (Figure S26, Supporting Information), hb‐P1‐8(1) NPs demonstrated dose‐dependent cytotoxicity to HeLa cells (Figure S27, Supporting Information). An IC_50_ value of 0.096 µg mL^−1^ was deduced, which is superior to those of free DOX (0.128 µg mL^−1^) and a reported polymer‐DOX prodrug (0.95 µg mL^−1^).^[^ ^32^ ^]^ These results indicate that hb‐poly(aminoacrylate)s obtained from hb‐PAs show great potential as polyprodrug amphiphiles for controlled drug release. In addition, our strategy could theoretically be applicable in constructing polyprodrug amphiphiles from our hb‐PAs and drugs bearing aliphatic amino groups.

### 2.7 Artificial Light‐Harvesting System

**Figure 6:**
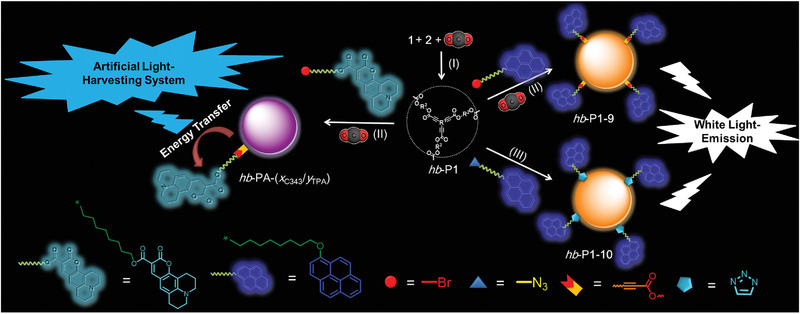
Construction of artificial light‐harvesting and white light‐emitting systems (I: Ag_2_WO_4_, Cs_2_CO_3_, DMAc, 80 °C, 1 h; II: Ag_2_WO_4_, Cs_2_CO_3_, DMAc, 100 °C, 12 h; III: Cu(PPh_3_)_3_Br, DMF, 60 °C, 12 h. I and II processed via a “one‐pot” tandem strategy; I and III processed via a “step‐by‐step” strategy.)

**Figure 7:**
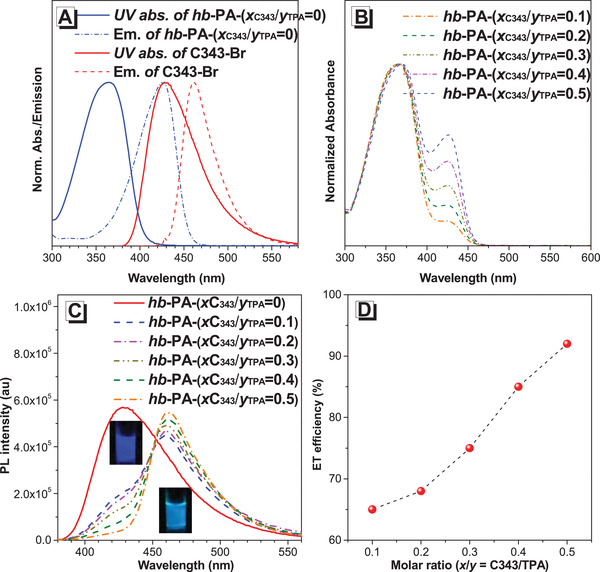
A) Normalized absorption and emission spectra of the donor hb‐PA‐(*x*_C343_/*y*_TPA_ = 0) and acceptor C343‐Br in THF. B) Normalized absorption spectra of hb‐PA‐(*x*_C343_/*y*_TPA_) in THF. C) Overlaid emission spectra of the hb‐PA‐(*x*_C343_/*y*_TPA_) in THF excited at *λ*_ex_ = 364 nm. Inset: photographs of hb‐PA‐(*x*_C343_/*y*_TPA_ = 0) and hb‐PA‐(*x*_C343_/*y*_TPA_ = 0.5) D) The ET efficiency of hb‐PA‐(*x*_C343_/*y*_TPA_) as a function of *x*/*y* = C343/TPA. Concentration: ≈1 × 10^−6^ m.

The high efficiency of our one‐pot tandem polymerization, the triphenylamine (TPA) moieties in the hb‐PAs, as well as natural photosynthesis and artificial light‐harvesting systems (LHSs)^[^ ^38^, ^39^, ^40^, ^41^, ^42^, ^43^, ^44^, ^45^, ^46^, ^47^, ^48^, ^49^, ^50^ ^]^ inspired investigations into further applications of these polymerizations. Therefore, we applied our strategy to constructing highly efficient, artificial LHSs by introducing light‐harvesting groups onto the peripheries of the polymers (**Figure** **6** and Scheme S5, Supporting Information). First, we modified a commercial dye, Coumarin 343 (C343), to C343‐Br to serve as an acceptor chromophore (Scheme S6, Supporting Information). The photo‐physical property investigation demonstrated that the TPA‐containing hb‐P1, in THF, when excited at 364 nm, displayed deep blue emission with a maximum wavelength of 428 nm. This largely overlaps with the absorption spectrum of C343‐Br, which peaks at 427 nm (**Figure** **7**A), and well meets the prerequisites necessary for an efficient energy transfer (ET). Meanwhile, C343‐Br showed weaker emission when excited at 364 nm than when it was excited at 427 nm, likely because of its low absorption at this wavelength (Figure 7A and Figure S28, Supporting Information).

Next, we polymerized CO_2_, 1, and 2 in the presence of Ag_2_WO_4_/Cs_2_CO_3_ for 1 h, followed by the addition of different amounts of C343‐Br. This produced hb‐PAs with different ratios of C343 and TPA moieties, namely hb‐PA‐(*x*_C343_/*y*_TPA_). These ratios of C343 and TPA were determined by ^1^H NMR spectra (Figures S29–S34, Supporting Information), and were almost consistent with the feeding ratios due to the highly efficient tandem reaction. The photo‐physical property measurements of hb‐PA‐(*x*_C343_/*y*_TPA_) showed two obvious peaks at 364 and 427 nm, assignable to the absorptions of the incorporated TPA‐based emissive units and C343 moieties, respectively (Figure 7B).

To study the ET efficiency of hb‐PA‐(*x*_C343_/*y*_TPA_), we varied the *x* value and kept the *y* value constant. As shown in Figure 7C,D, increasing the ratio of *x*/*y* enhanced the ET efficiency and a value of 92% was achieved when the ratio was 0.5 (see the Supporting Information for the details), which is comparable to those of previous reports.^[^ ^38^, ^43^ ^]^ When hb‐PA‐(*x*_C343_/*y*_TPA_) with an *x*/*y* ratio of 0.5 was excited at 364 nm, no emission peak of the TPA‐based emissive unit at 428 nm was recorded. Instead, a strong emission peak from C343 was seen at 462 nm, indicative of an efficient ET from the TPA‐based emissive unit to the C343 chromophores within one polymer. Furthermore, when this polymer was excited at a wavelength of 427 nm, the emission intensity were weaker than when the polymer was excited at 364 nm, further confirming the light‐harvesting effect of this hyperbranched polymer (Figure S35, Supporting Information).

### 2.8 White Light‐Emitting Polymers

We also constructed white light‐emitting (WLE) polymeric materials by eliminating the ET through altering the grafted periphery moiety from C343 to pyrene derivatives, whose emission is complementary to that of hb‐P1 in the solid state.^[^ ^51^, ^52^, ^53^, ^54^, ^55^ ^]^

As shown in Scheme S7 (Supporting Information), the pyrene‐based, deep‐blue emitters 13 and 14 were first synthesized in excellent yields. We then studied the absorption and photoluminescence (PL) spectra of 13, 14, and hb‐P1 in their film states (Figure S36, Supporting Information). The absorption and PL peaks of hb‐P1 in the solid state are located at 372 and 578 nm, respectively, indicating that this polymer possesses a large Stokes shift; whereas, the PL peaks of 13 and 14 in the solid states are both recorded at 460 nm. These results show that there is only a small overlap between the absorption of hb‐P1 and the emission of 13 and 14, efficiently avoiding ET from the excited states of 13 and 14 to the ground state of hb‐P1 and facilitating the fabrication of WLE polymers.

Encouraged by these results, we synthesized hb‐P1‐9 by our one‐pot tandem polymerization, using CO_2_, 1, 2, and 13 (Figure 6 and Figure S37 and Scheme S8, Supporting Information). Moreover, we prepared hb‐P1‐10 by the functionalization of hb‐P1 with 14 via a Cu(I)‐catalyzed azide–alkyne cycloaddition (Figure 6 and Figure S38 and Scheme S9, Supporting Information). As expected, in the solid state both hb‐P1‐9 and hb‐P1‐10 emit broadly, and their PL profiles cover the entire visible light range. Furthermore, the *Φ*_F_ values and CIE chromaticity coordinates for hb‐P1‐9 were measured to be 10.6% and (0.31, 0.32), and 6.9% and (0.33, 0.33), respectively, for hb‐P1‐10, clearly demonstrating WLE properties (Figures S39 and S40,Supporting Information) that are comparable to those in previous reports.^[^ ^56^, ^57^, ^58^, ^59^ ^]^

## 3 Conclusion

CO_2_‐based hb‐PAs with high *M*_w_ (up to 226 700 g mol^−1^) and DB values were facilely prepared under atmospheric pressure in only 3 h. The resultant polymers are soluble in commonly used organic solvents and thermally stable, and the TPE/TPP‐containing hb‐PAs show typical AIE features. Because they possess two types of ethynyl groups with different reactivities, these hb‐PAs can undergo site‐selective, three‐step functionalizations with nearly 100% conversion in each step. Furthermore, through a simple, one‐pot, two/three‐step, four/five‐component tandem polymerization strategy, the hb‐PAs can be selectively tailored to contain hydrophilic OEG chains in their branched chains, on their peripheries, or both. Taking advantage of this simple and versatile platform for site‐selective functionalizations, hyperbranched polyprodrug amphiphiles were generated with high drug (DOX) loading content (44.3 wt%), excellent, sustained acid‐targeted release, and higher in vitro anti‐cancer efficacy than free DOX. By controlling the ET in hb‐PAs, an artificial light‐harvesting system with high ET efficiency (up to 92%) and white light‐emitting hb‐PAs were also constructed. Thus, this work not only provides a new pathway to convert CO_2_ into HBPs, but also presents a versatile platform for site‐selective multistep functionalizations toward functional materials.

## Conflict of Interest

The authors declare no conflict of interest.

## Supporting information

Supporting Information
