# Supplementary material for: Site‐Selective, Multistep Functionalizations of CO2‐Based Hyperbranched Poly(alkynoate)s toward Functional Polymetric Materials
Source: Adv Sci (Weinh). 2020 Jul 8;7(17):2000465. doi: 10.1002/advs.202000465 (PMC7507432; doi:10.1002/advs.202000465)
Supplement: Supplementary file 1 — Supporting Information [file ADVS-7-2000465-s001.pdf]

Supporting Information

Site-Selective Multi-Step Functionalizations of CO_2-Based Hyperbranched Poly(alkynoate)s Toward Functional Polymetric Materials

Bo Song, Rongyuan Zhang, Rong Hu, Xu Chen, Dongming Liu, Jiali Guo, Xiaotian Xu, Anjun Qin, and Ben Zhong Tang**

Dr. B. Song, R. Zhang, Dr. R. Hu, X. Chen, D. Liu, J. Guo, X. Xu, Prof. A. Qin, Prof. B. Z. Tang
State Key Laboratory of Luminescent Materials and Devices, Guangdong Provincial Key Laboratory of Luminescence from Molecular Aggregates, Center for Aggregation-Induced Emission, Guangzhou International Campus, South China University of Technology, Guangzhou 510640, China
E-mail: msqinaj@scut.edu.cn

Prof. B. Z. Tang
Department of Chemistry, Hong Kong Branch of Chinese National Engineering Research Centre for Tissue Restoration and Reconstruction, Institute for Advanced Study, and Department of Chemical and Biological Engineering, The Hong Kong University of Science & Technology, Clear Water Bay, Kowloon, Hong Kong, China.
E-mail: tangbenz@ust.hk

R. Zhang
Department of Urology, The First Affiliated Hospital of Soochow University, 188 Shizi RD, Suzhou 215006, China

Contents

Materials and instruments S3

Preparation of monomer **3b** S4

Optimization of polymerization conditions S5

Typical procedures for polymerization of CO_2, **1** and **2** S6

Characterization data for hyperbranched poly(alkynoate)s S7

GPC curves for hyperbranched poly(alkynoate)s S8

TGA curves of hyperbranched poly(alkynoate)s S9

Preparation of model compounds S9

FT-IR and NMR spectra of monomers and polymers S11

Degree of Branching S17

Photophysical properties S18

Procedures of site-selective three-step functionalizations of *hb*-P**1** S19

Procedures of site-selective tandem polymerization S21

Synthesis of hyperbranched polyprodrug amphiphiles S23

Preparation of hyperbranched polyprodrug NPs S25

In vitro drug release S26

CLSM study S27

In vitro anti-cancer efficacy S27

Construction of artificial light-harvesting system S28

Construction of white light- emitting system S35

References S38

Materials and instruments

All manipulations involving air- and/or water-sensitive compounds were carried out in a glove box or with the standard Schlenk techniques. CO_2 (99.995%) was purchased from Guangzhou Ke Ming Gases Co., Ltd. and used as received. Monomers **1** and **3a** were prepared according to our previous reported procedures.[1] 1,8-Dibromooctane, Ag_2WO_4, Cs_2CO_3, *N*,*N*-dimethylacetamide (DMAc), benzylamine, 1-bromooctane, morpholine, methyl-PEG3-amine, 1-bromo-2-(2-methoxyethoxy)ethane, mPEG-Br (M_n = 2000 Da), 1-butyl iodide, Doxorubicin (DOX), coumarin 343 (C343) and 1-hydroxypyrene were purchased from Energy Chemical, Sigma-Aldrich, TCI, Aladdin and used without further purification. HeLa cells were obtained from cell culture center of Institute of Basic Medical Sciences, Chinese Academy of Medical Science (Beijing, China). PBS buffer was purchased from Thermo Fisher Scientific (Shanghai, China). Lysotracker DND 99, LysoTracker DND-26 and Hoechst 33342 were purchased from Thermo Fisher Scientific (Shanghai, China). Water was purified with a Millipore filtration system. Methyl thiazolyl tetrazolium (MTT) was purchased from Tiangen Biotech (Beijing, China).

^{1}H and ^{13}C NMR spectra were measured on a Bruker Avance 500 MHz NMR spectrometer using deuterated dichloromethane as solvent and tetramethylsilane (TMS, δ = 0) as internal reference. Fourier transform infrared (FT-IR) spectra were measured on a Bruker Vector 33 FT-IR spectrometer (KBr disk). High resolution mass spectrometry (HRMS) measurements were performed on a GCT premier CAB048 mass spectrometer operating in MALDI-TOF mode. The number- (M_n) and weight-average (M_w) molecular weights and polydispersity indices ($Đ = M_w/M_n$) of polymers were estimated by gel permeation chromatography (GPC) system, and tetrahydrofuran (THF) was used as eluent at a flow rate of 0.5 mL/min. A set of monodispersed linear polystyrenes covering the M_w range of 10^3–10^7 g/mol were utilized as standards. Absolute molecular weights were measured by a DAWN multi-angle laser light scattering (MALLS) detector, and an Optilab T-rEX differential refractometer (both from Wyatt Technology). (*d*n/*d*c of *hb*-PAs in THF = 0.1875 mL/g). *In-situ* FT-IR was measured on METTLER TOLEDO ReactIR™ 15. Thermogravimetric analysis was carried out on a SHIMADZU TGA-50

analyzer under a nitrogen atmosphere at a heating rate of 20 K/min. UV−vis absorption spectra were recorded on a SHIMADZU UV-2600 spectrophotometer. Fluorescence spectra were recorded on a Horiba Fluoromax-4 fluorescence spectrophotometer. Absolute fluorescence quantum yields and CIE chromaticity coordinates were measured using a Hamamatsu absolute PL quantum yield spectrometer C11347 Quantaurus QY. pH values were measured via METTLER TOLEDO FiveEasy FE20. Confocal laser scanning microscope (CLSM) characterization was conducted with a confocal laser scanning biological microscope (LSM 710, Zeiss, Germany). The absorbance for MTT analysis was recorded on a microplate reader (Thermo Fisher, USA) at a wavelength of 570 nm. Size measurements were conducted on Dynamic Light Scattering (ZSE, Malvern, UK). Transmission electron microscope was carried on JEM-2100F (JEOL, Japan).

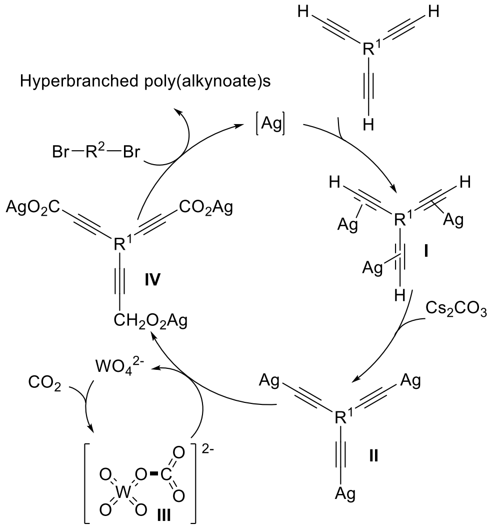

Scheme S1. Proposed mechanism of Ag_2WO_4-catalyzed polymerization of CO_2, triyne and alkyl dihalide.

Preparation of monomer 3b

The monomer **3b** was synthesized in two steps, and the synthetic routes are show in Scheme S2.

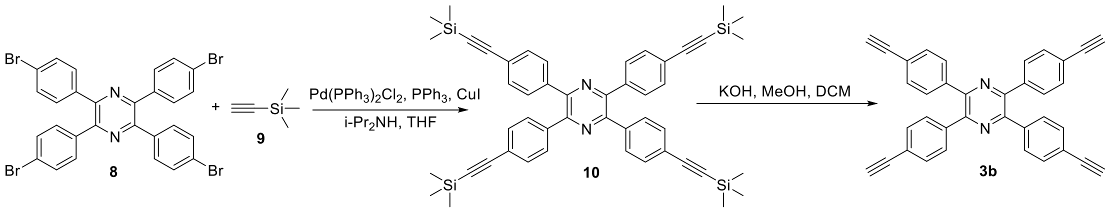

Scheme S2. Synthetic routes to monomer **3b**.

2,3,5,6-Tetrakis(4-((trimethylsilyl)ethynyl)phenyl)pyrazine (10): Into a 250 mL round-bottom flask were added $PdCl_2(PPh_3)_2$ (140 mg, 0.2 mmol), CuI (76 mg, 0.4 mmol), PPh_3 (52 mg, 0.2 mmol), **8** (1.400 g, 2 mmol), and a mixture of THF/*i*-Pr_2NH (50:50 v/v) (100 mL) under nitrogen. After the catalysts were completely dissolved, trimethylsilylacetylene (**9**, 1.7 mL, 12 mmol) was injected. The solution was stirred at 80 ℃ for 2 days, and then the formed precipitates were removed by filtration and washed with diethyl ether. The filtrate was concentrated by a rotary evaporator under reduced pressure, and the crude product was purified by a silica gel column chromatography using PE/DCM (4:1 v/v) as eluent. Yellow powder of **10** was obtained in 65% yield (1.0 g).

2,3,5,6-Tetrakis(4-ethynylphenyl)pyrazine (3b): Into a 250 mL round-bottom flask was added **10** (1.0 g, 1.3 mmol) and DCM (60 mL). Then KOH (875 mg, 15.6 mmol) dissolved in methanol (60 mL) was added. The mixture was stirred at room temperature overnight. After most of the solvent was evaporated, 1 M HCl solution (50 mL) was added. The aqueous solution was extracted with DCM for three times. The organic phases were combined and washed with water and brine and then dried over $MgSO_4$ for 1 h. After filtration and solvent evaporation, the crude product was purified by a silica gel column chromatography using PE/DCM (4:1 v/v) as eluent. Yellow powder of **3a** was obtained in 91% yield (569 mg). ^1H NMR (500 MHz, CD_2Cl_2), δ (TMS, ppm): 7.61-7.56 (m, 8H), 7.49-7.45 (m, 8H), 3.22 (s, 4H). ^{13}C NMR (125 MHz, CD_2Cl_2), δ (TMS, ppm): 148.26, 138.81, 132.51, 130.21, 123.10, 83.48, 78.92. HRMS (MALDI-TOF): m/z 480.1633 (M^+, calcd 480.1626).

Optimization of polymerization conditions

Table S1. Effect of monomer mole ratio on the polymerization of **1**, **2** and CO_2 [a]

entry	[**1**]/[**2**]	yield (%)	S[b]	M_w[c]	PDI[c]
1	1:1	95	√	20 800	2.18
2	2:3		gelled		

[a] Carried out in *N*,*N*-dimethylacetamide (DMAc) at 80 °C under CO_2 (balloon) for 3 h in the presence of Ag_2WO_4 and Cs_2CO_3. [**1**] = 0.20 M, [**1**]/[Ag_2WO_4]/[Cs_2CO_3] = 1:0.15:9. [b] Solubility (S) tested in commonly used organic solvents such as THF and DCM: √ = completely soluble. [c] Estimated by gel permeation chromatography (GPC) in THF on the basis of a polystyrene calibration. PDI = polydispersity index (M_w/M_n, M_w = weight-average molecular weight, M_n = number-average Molecular Weight).

The yield of the product was calculated via following equation:

$$\text{Yield} = \frac{m_a}{m_t} \times 100\% \quad \text{(S1)}$$

where m_t is the theoretical mass of polymers we could obtain, m_a is actual mass of polymers we got. Taking "Table S1, entry 1" for example, 0.2 mmol **1**, **2** and CO_2 were used to generate polymer. The molar ratio of **1** and **2** is 1:1. Thus, **2** was completely consumed theoretically and theoretical mass of polymers should be [(317.4 - 2) + 44.0×2 + 112.2]×0.2 = 103.1 mg, where 317.4, 44.0 and 112.2 are the relative molecular mass of **1**, CO_2 and C_8H_{16}. The actual mass of polymers we got is 97.9 mg. Thus, the yield is 97.9/103.1 = 95%.

Table S2. Time course on the polymerization of **1**, **2** and CO_2 [a]

entry	t (h)	yield (%)	M_w [b]	PDI [b]
1	1	76	8 500	1.45
2	2	87	14 100	1.86
3	3	92	21 500	2.21
4	4	93	22 100	2.24

[a] Carried out in DMAc at 80 ^oC under CO_2 (balloon) in the presence of Ag_2WO_4 and Cs_2CO_3. [**1**] = [**2**] = 0.20 M, [**1**]/[**2**]/[Ag_2WO_4]/[Cs_2CO_3] = 1:1:0.15:9. [b] Estimated by GPC in THF on the basis of a polystyrene calibration. PDI = polydispersity index (M_w/M_n, M_w = weight-average molecular weight, M_n = number-average molecular weight).

Table S3. Polymerization results of different monomers[a]

entry	monomer	polymer	yield(%)	$M_{w,\,GPC}$ [b]	PDI [b]	$M_{w,\,MALLS}$ [c]	PDI [c]
1	**1** + **2** + CO_2	*hb*-P**1**	90	21 400	2.13	226 700	2.27
2	**3a** + **2** + CO_2	*hb*-P**2**	71	11 100	1.70	27 100	2.19
3	**3b** + **2** + CO_2	*hb*-P**3**	79	7 800	1.33	15 400	1.98

[a] Carried out in DMAc at 80 ^oC under CO_2 (balloon) for 3 h in the presence of Ag_2WO_4 and Cs_2CO_3. [$\mathbf{A_n}$] = [**2**] = 0.30 M, [$\mathbf{A_n}$]/[**2**]/[Ag_2WO_4]/[Cs_2CO_3] = 1:1:0.15:6. [b] Estimated by GPC in THF on the basis of a polystyrene calibration. PDI = polydispersity index (M_w/M_n, M_w = weight-average molecular weight, M_n = number-average molecular weight). [c] Determined by GPC-MALLS analysis in THF.

Typical procedures for polymerization of CO_2, 1 and 2

A typical procedure of the polymerization of **1**, **2** and CO_2 is given below as an example. Into a 10 mL dried Schlenk tube equipped with magnetic stirrer was placed with **1** (63.5 mg, 0.2 mmol), **2** (54.4 mg, 0.2 mmol), Ag_2WO_4 (13.9 mg, 0.03 mmol), Cs_2CO_3 (391.0 mg, 1.2 mmol) under CO_2 (balloon). Dried DMAc (0.67 mL) were injected into the tube by syringes. The resultant mixture was stirred at 80 ^oC under atmospheric pressure for 3 h. After cooled to room temperature, 4 mL of DCM was added to

dilute the mixture. Then the solution was added dropwise into 200 mL of methanol via a cotton filter under stirring. The precipitate was allowed to stand overnight and then collected by filtration. The polymer was washed with methanol and dried under vacuum at room temperature to a constant weight.

Characterization data for hyperbranched poly(alkynoate)s

hb-P**1**: A yellow solid was obtained in 90% yield (Table S3, entry 1). FT-IR (KBr disk), υ (cm^{-1}): 3287, 3045, 2932, 2854, 2208, 2104, 1708, 1592, 1501, 1388, 1322, 1290, 1272, 1194, 1169, 1015, 835, 770, 747, 724, 567, 543. ^{1}H NMR (500 MHz, CD_2Cl_2), δ (TMS, ppm): 7.57-7.36, 7.12-6.96, 4.19, 3.14, 3.13, 1.70, 1.37. ^{13}C NMR (125 MHz, CD_2Cl_2), δ (TMS, ppm): 154.48, 154.42, 154.37, 149.27, 148.85, 148.51, 147.06, 146.72, 134.92, 134.83, 134.75, 133.97, 133.83, 125.68, 125.12, 124.69, 124.02, 123.17, 118.61, 117.97, 115.01, 114.28, 113.39, 86.59, 86.27, 86.01, 83.52, 83.39, 81.32, 81.18, 81.04, 77.76, 77.51, 66.53, 66.50, 66.45, 29.46, 28.83, 26.15.

hb-P**2**: A yellow solid was obtained in 71% yield (Table S3, entry 2). FT-IR (KBr disk), υ (cm^{-1}): 3287, 3073, 3034, 2932, 2856, 2219, 2107, 1709, 1601, 1503, 1463, 1436, 1402, 1388, 1291, 1195, 1172, 1110, 1018, 978, 870, 839, 817, 747, 709, 651, 574, 541. ^{1}H NMR (500 MHz, CD_2Cl_2), δ (TMS, ppm): 7.43-7.19, 7.08-6.88, 4.18, 3.13, 3.12, 1.68, 1.36. ^{13}C NMR (125 MHz, CD_2Cl_2), δ (TMS, ppm): 154.22, 145.73, 145.50, 145.45, 145.43, 145.22, 145.15, 143.56, 143.52, 143.50, 143.31, 143.24, 143.04, 142.60, 142.24, 141.96, 141.62, 141.27, 140.98, 140.65, 133.05, 132.99, 132.94, 132.92, 132.23, 132.21, 132.18, 132.16, 132.13, 132.10, 131.88, 131.86, 131.64, 131.62, 121.54, 121.40, 121.34, 121.25, 121.19, 118.80, 118.75, 118.65, 118.60, 118.44, 85.85, 85.76, 85.68, 83.63, 83.61, 83.57, 83.55, 81.62, 81.56, 81.49, 78.37, 78.27, 78.18, 78.15, 66.57, 29.45, 28.80, 26.13..

hb-P**3**: A yellow solid was obtained in 79% yield (Table S3, entry 3). FT-IR (KBr disk), υ (cm^{-1}): 3287, 3047, 2932, 2854, 2219, 2104, 1709, 1600, 1557, 1506, 1462, 1411, 1388, 1290, 1194, 1174, 1113, 1096, 1010, 932, 843, 779, 746, 697, 658, 628, 547. ^{1}H NMR (500 MHz, CD_2Cl_2), δ (TMS, ppm): 7.69-7.41, 4.21, 3.22, 1.71, 1.38. ^{13}C NMR (125 MHz, CD_2Cl_2), δ (TMS, ppm): 154.19, 148.00, 140.44, 138.54, 133.34, 133.30, 132.57, 132.56, 132.52, 130.43, 130.22, 123.39, 120.70, 85.41, 83.39, 82.15, 79.04, 66.68, 29.47, 28.82, 26.15.

GPC curves for hyperbranched poly(alkynoate)s

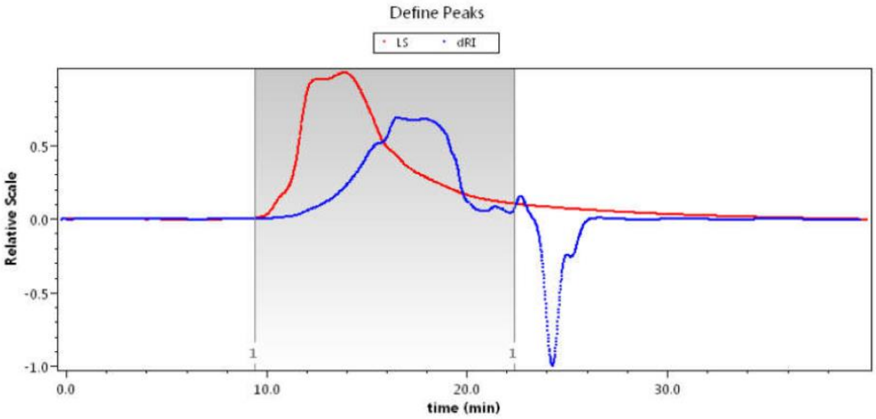

Figure S1. GPC curve of *hb*-P**1** (red line: light scattering signal; blue line: refractive index signal).

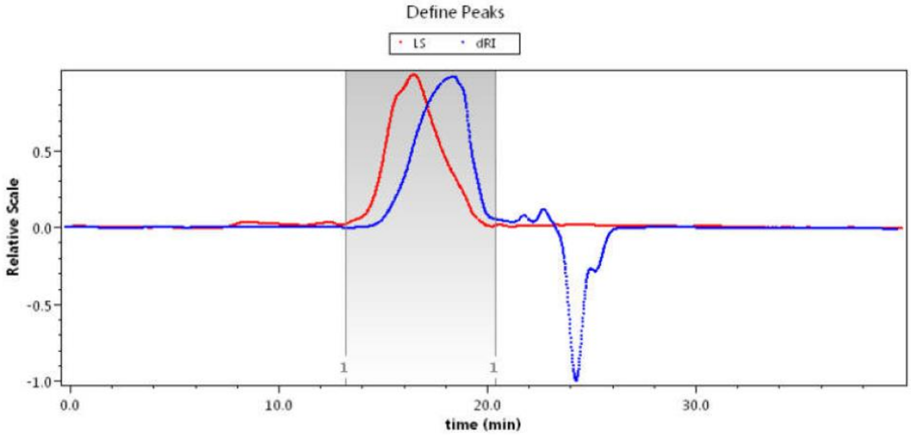

Figure S2. GPC curve of *hb*-P**2** (red line: light scattering signal; blue line: refractive index signal).

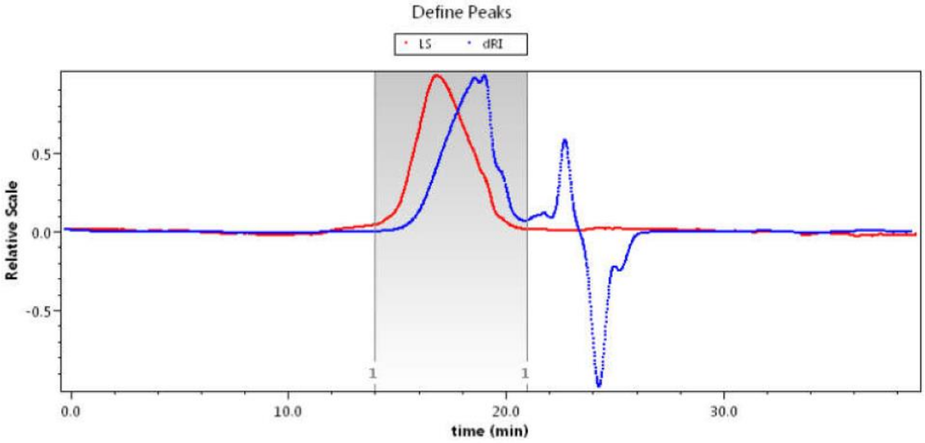

Figure S3. GPC curve of *hb*-P**3** (red line: light scattering signal; blue line: refractive index signal).

TGA curves of hyperbranched poly(alkynoate)s

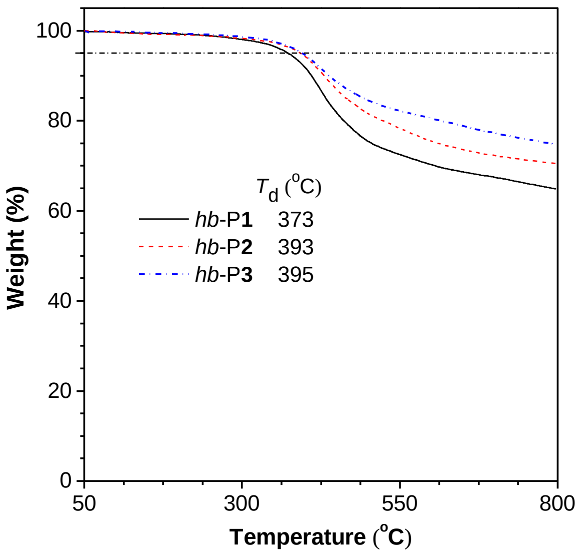

Figure S4. TGA thermograms recorded under nitrogen at a heating rate of 20 °C min^{-1}.

Preparation of model compounds

Model compounds **5–7** was synthesized by three-component reaction of **1,** 1-bromooctane and CO_2. Typically, into a 10 mL dried Schlenk tube equipped with magnetic stirrer was placed with **1** (158.7 mg, 0.5 mmol), 1-bromooctane (193.1 mg, 1 mmol), Ag_2WO_4 (34.8 mg, 0.075 mmol), and Cs_2CO_3 (977.5 mg, 3 mmol) under CO_2 (balloon). Dried DMAc (3 mL) were injected into the tube by a syringe. The resultant mixture was stirred at 80 °C under atmospheric CO_2 for 3 h. Then the reaction mixture was cooled to room temperature and extracted with dichloromethane (DCM) (60 mL × 3). The organic layer was washed by water (100 mL × 3) and dried over Na_2SO_4. After filtration, the filtrate was concentrated and purified by silica gel column chromatography using petroleum ether (PE)/DCM mixture as the eluent, a yellow solid of compounds **5–7** (293.1 mg) was obtained.

5: FT-IR (KBr disk), υ (cm^{-1}):3287, 3039, 2957, 2927, 2854, 2211, 2107, 1708, 1612, 1598, 1502, 1466, 1391, 1320, 1292, 1266, 1209, 1190, 1174, 1105, 1018, 834, 805, 769, 744, 725, 645, 568, 560, 542. ^{1}H NMR (500 MHz, CD_2Cl_2), δ (TMS, ppm): 7.49-7.39 (m, 6H), 7.07-7.00 (m, 6H), 4.19 (t, J = 5.4 Hz, 2H), 3.13 (s, 2H), 1.74-1.63 (m, 2H), 1.43-1.20 (m, 10H), 0.88 (t, J = 5.6 Hz, 3H).. ^{13}C NMR (125 MHz, CD_2Cl_2), δ (TMS, ppm): 154.50, 149.27, 147.08, 134.74, 133.83, 125.12, 123.19, 117.96, 113.43, 86.54, 83.52, 81.06, 77.48, 66.54, 32.20, 29.59, 29.58, 28.89, 26.25, 23.06, 14.27. HRMS (MALDI-TOF): m/z

473.2325 (M^+, calcd 473.2355).

6: FT-IR (KBr disk), υ (cm^{-1}):3287, 3039, 2957, 2927, 2854, 2211, 2107, 1708, 1592, 1502, 1466, 1384, 1320, 1292, 1274, 1194, 1166, 1006, 838, 769, 744, 725, 676, 645, 566, 542. ^1H NMR (500 MHz, CD_2Cl_2), δ (TMS, ppm): 7.51-7.42 (m, 6H), 7.08-7.04 (m, 6H), 4.19 (t, J = 5.4 Hz, 4H), 3.14 (s, 1H), 1.74-1.63 (m, 4H), 1.43-1.23 (m, 20H), 0.88 (t, J = 5.6 Hz, 6H). ^{13}C NMR (125 MHz, CD_2Cl_2), δ (TMS, ppm): 154.44, 148.85, 146.75, 134.82, 133.96, 125.67, 124.03, 118.59, 114.31, 86.22, 83.39, 81.19, 77.72, 66.58, 32.20, 29.59, 29.58, 28.88, 26.25, 23.06, 14.27. HRMS (MALDI-TOF): m/z 629.3474 (M^+, calcd 629.3505).

7: FT-IR (KBr disk), υ (cm^{-1}):3062, 2957, 2927, 2854, 2211, 1708, 1592, 1502, 1463, 1382, 1320, 1292, 1274, 1194, 1166, 838, 769, 744, 725, 571, 543. ^1H NMR (500 MHz, CD_2Cl_2), δ (TMS, ppm): 7.55-7.49 (m, 6H), 7.12-7.06 (m, 6H), 4.19 (t, J = 5.4 Hz, 6H), 1.74-1.64 (m, 6H), 1.43-1.22 (m, 30H), 0.89 (t, J = 5.6 Hz, 9H). ^{13}C NMR (125 MHz, CD_2Cl_2), δ (TMS, ppm): 154.39, 148.52, 134.91, 124.69, 115.03, 85.96, 81.32, 66.62, 32.20, 29.59, 29.58, 28.88, 26.24, 23.06, 14.27. HRMS (MALDI-TOF): m/z 785.4639 (M^+, calcd 785.4655).

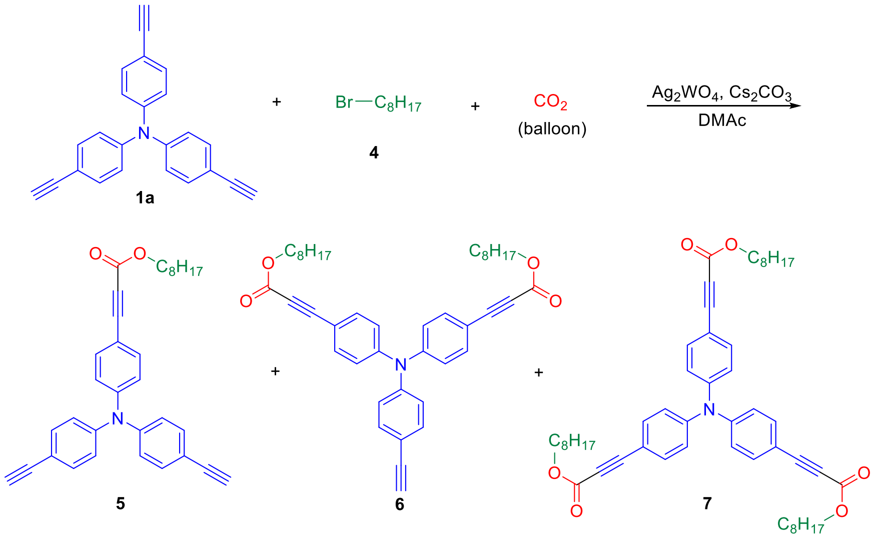

Scheme S3. Synthetic route to model compounds **5-7**.

FT-IR and NMR spectra of monomers and polymers

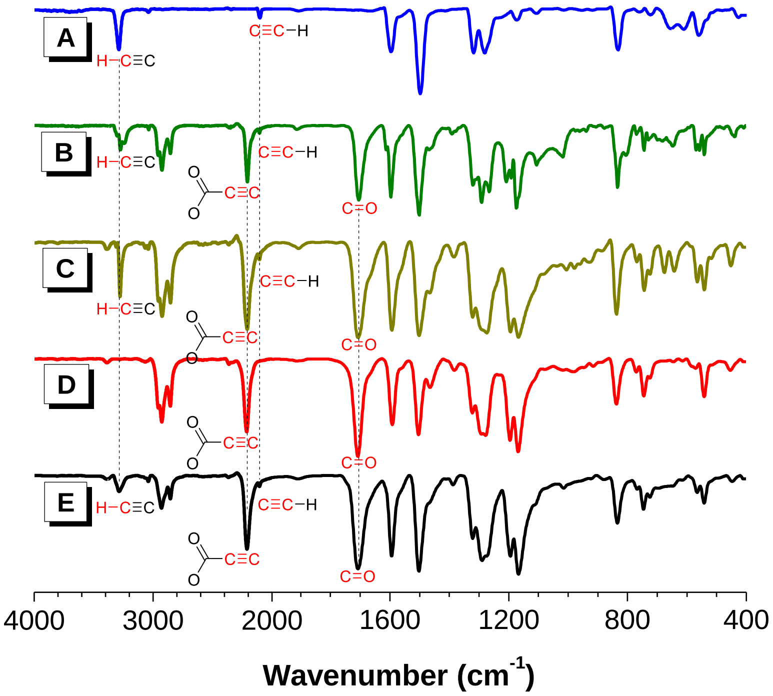

Figure S5. FT-IR spectra of monomer **1** (A), model compounds **5** (B), (C) **6**, (D) **7** and polymer *hb*-P**1** (E).

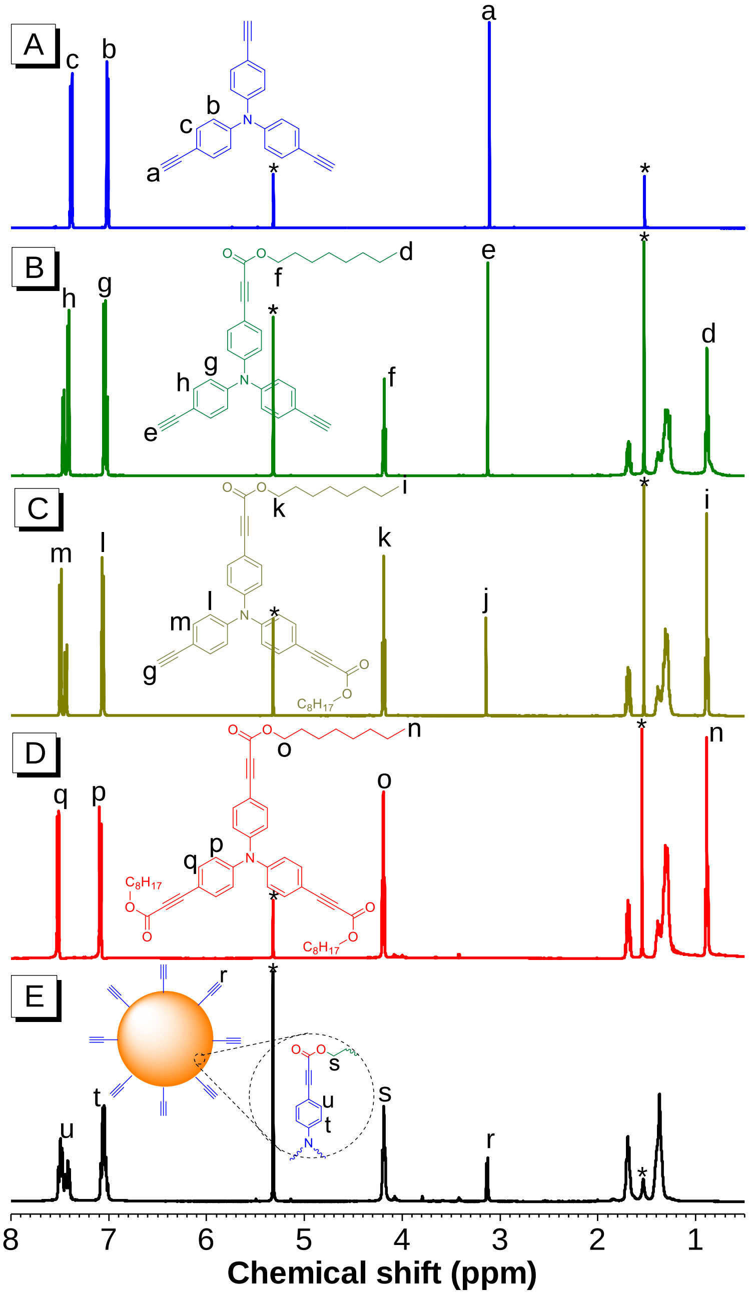

Figure S6. ^{1}H NMR spectra of monomer **1** (A), model compounds **5** (B), (C) **6**, (D) **7** and polymer *hb*-P**1** (E) in DCM-d_2. The solvent peaks are marked with asterisks.

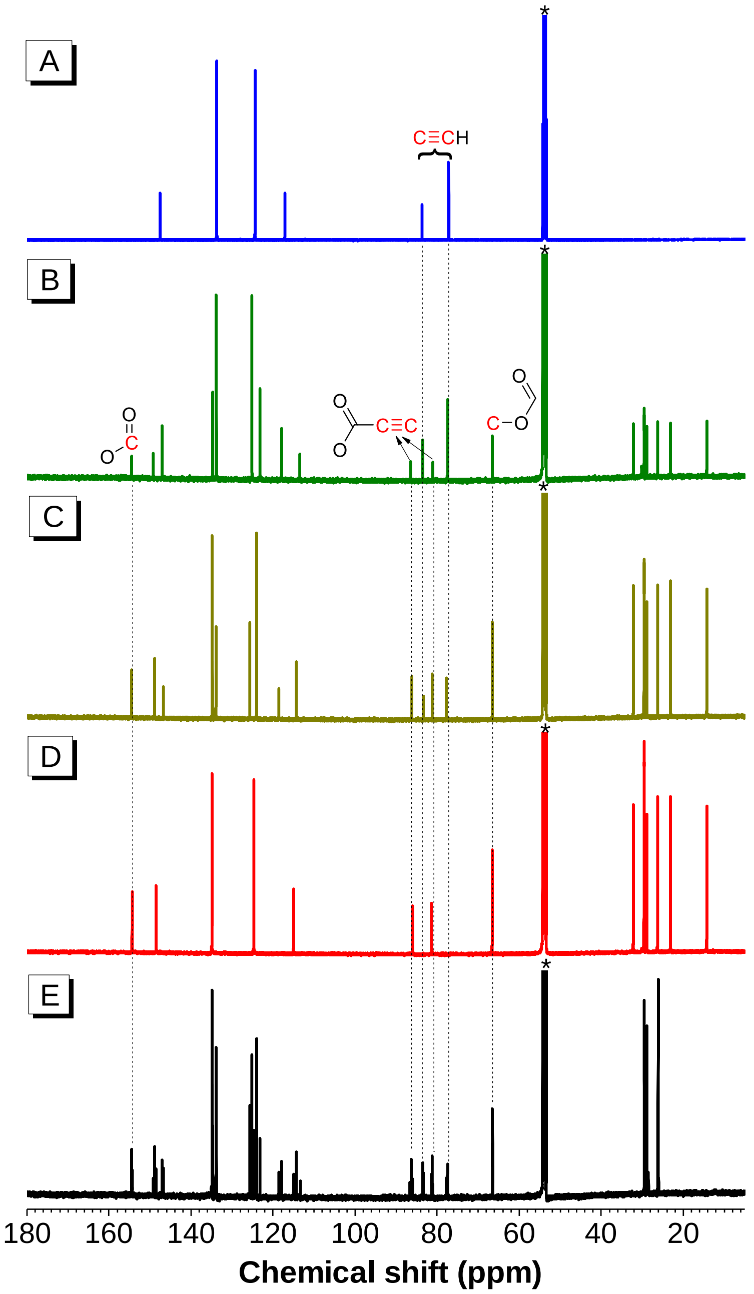

Figure S7. ^{13}C NMR spectra of monomer **1** (A), model compounds **5** (B), (C) **6**, (D) **7** and polymer *hb*-**P1** (E) in DCM-d_2. The solvent peaks are marked with asterisks.

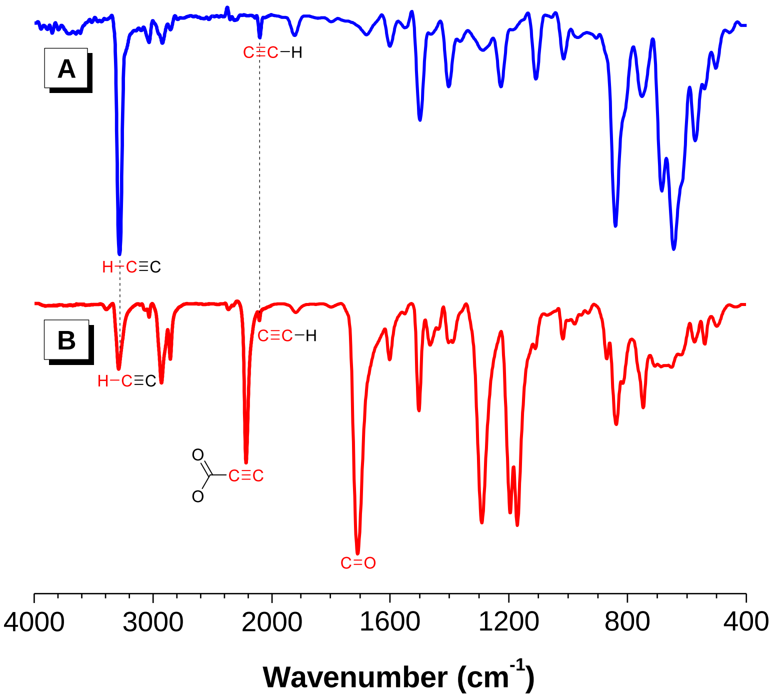

Figure S8. FT-IR spectra of (A) monomer **3a** and (B) polymer *hb*-P**2**.

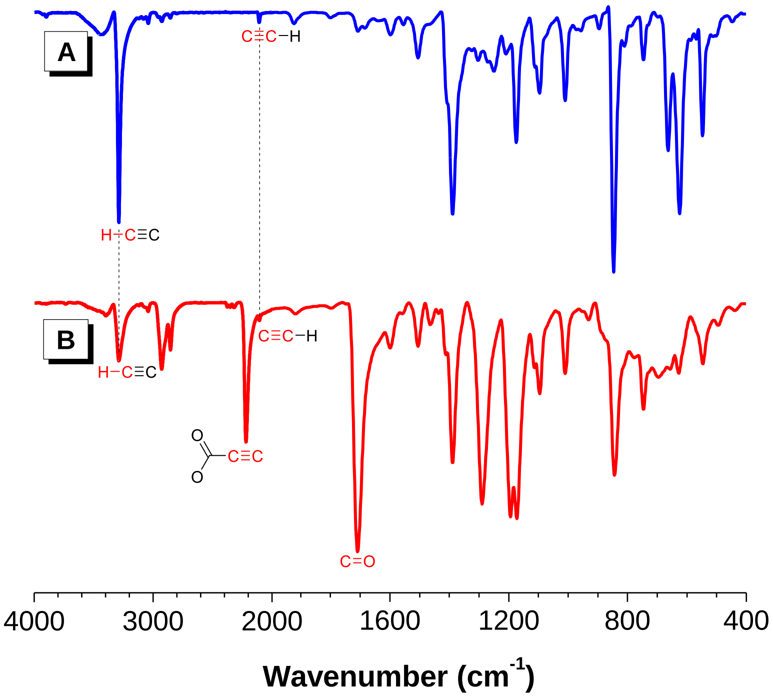

Figure S9. FT-IR spectra of (A) monomer **3b** and (B) polymer *hb*-P**3**.

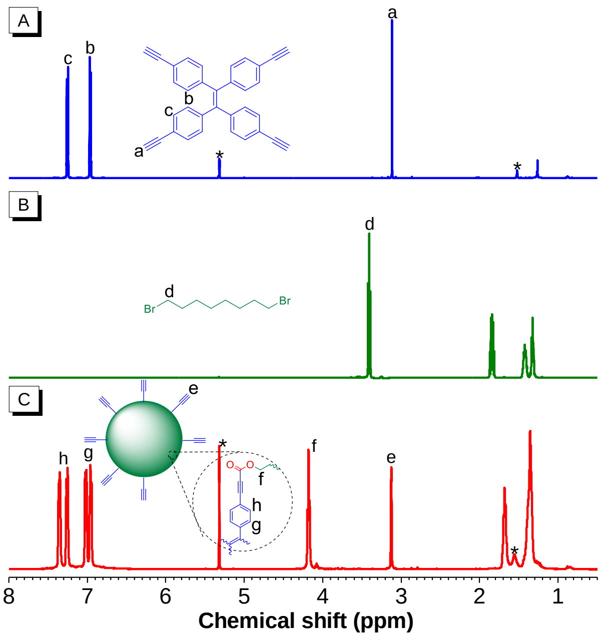

Figure S10. ^{1}H NMR spectra of (A) monomer **3a**, (B) monomer **2** and (C) polymer *hb*-P**2** in DCM-d_2. The solvent peaks are marked with asterisks.

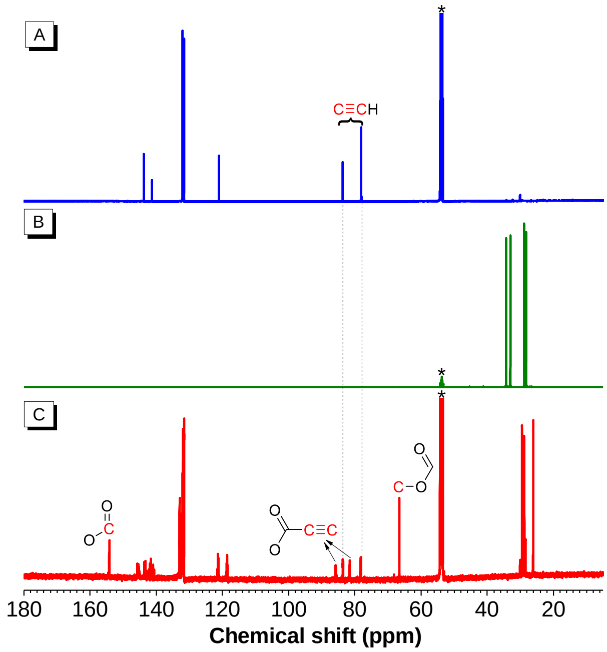

Figure S11. ^{13}C NMR spectra of (A) monomer **3a**, (B) monomer **2** and (C) polymer *hb*-P**2** in DCM-d_2. The solvent peaks are marked with asterisks.

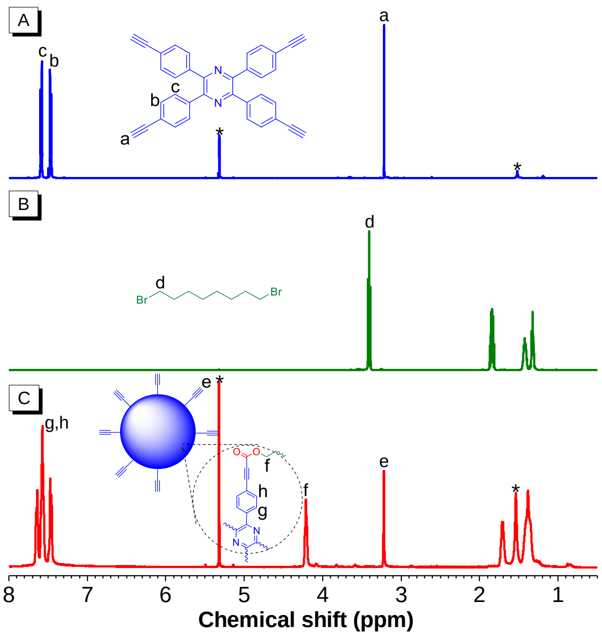

Figure S12. ^{1}H NMR spectra of (A) monomer **3b**, (B) monomer **2** and (C) polymer *hb*-P**3** in DCM-d_2. The solvent peaks are marked with asterisks.

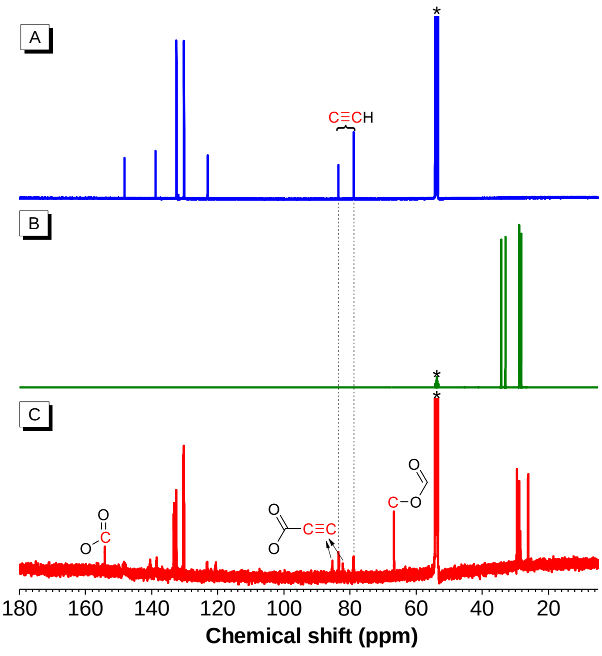

Figure S13. ^{13}C NMR spectra of (A) monomer **3b**, (B) monomer **2** and (C) polymer *hb*-P**3** in DCM-d_2. The solvent peaks are marked with asterisks.

Degree of Branching

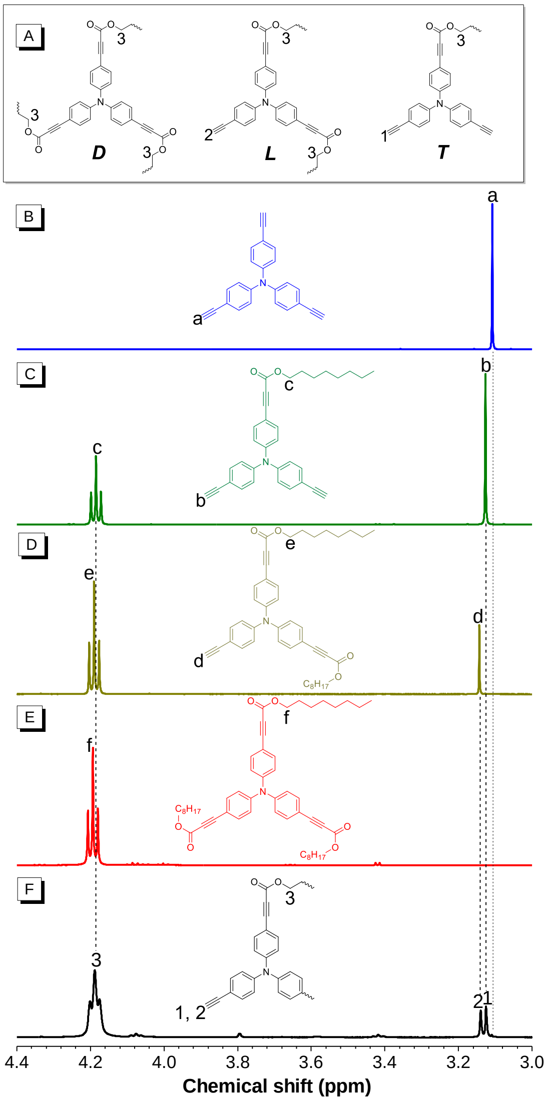

Figure S14. (A) Chemical structures of dendritic (*D*), linear (*L*), and terminal (*T*) units in *hb*-P**1** and ^{1}H NMR spectra of monomer **1** (B), model compounds **5** (C), **6** (D), **7** (E), and polymer *hb*-P**1** (F) in DCM-d_2. The solvent peaks are marked with asterisks.

As shown in Figure S16A, there are three structural components in *hb*-P**1**: dendritic (*D*), linear (*L*), and terminal (*T*) units. By comparing the ^{1}H NMR spectrum of *hb*-P**1** with those of its monomer **1** and model compounds **5**, **6** and **7** (Figure S14B-F), we can conclude that two ethynyl proton resonance peaks of *hb*-P**1** are corresponding with these of terminal and linear units. Thus, the following relationships among the contents or fractions (*f*) of the structural units can be established.

$$f_T + f_L + f_D = 1 \text{ (S2)}$$

$$\frac{2f_T}{f_L} = \frac{A_1}{A_2} \quad (S3)$$

$$\frac{2f_T}{2f_T + 4f_L + 6f_D} = \frac{A_1}{A_3} \quad (S4)$$

where A_1, A_2 and A_3 represent the integrals of the areas of resonance peaks 1, 2 and 3, respectively, as labelled in Figure S14F. The values can be determined from the ^{1}H NMR spectral data, from which the following equations are deduced:

$$\frac{2f_T}{f_L} = \frac{1.26}{1} \quad (S5)$$

$$\frac{2f_T}{2f_T + 4f_L + 6f_D} = \frac{0.116}{1} \quad (S6)$$

From the above equations, f_D, f_L and f_T were calculated to be:

$$f_D = 0.36 \quad f_L = 0.39 \quad f_T = 0.25 \quad (S7)$$

According to the definition, DB is expressed as:

$$\mathrm{DB} = \frac{f_D + f_T}{f_D + f_L + f_T} \quad (S8)$$

Incorporating the numbers in equation 9 into equation 10 yielded the DB value of *hb*-P**1**:

$$\mathrm{DB} = 1 - f_L = \ 0.61 \quad (S9)$$

Photophysical properties

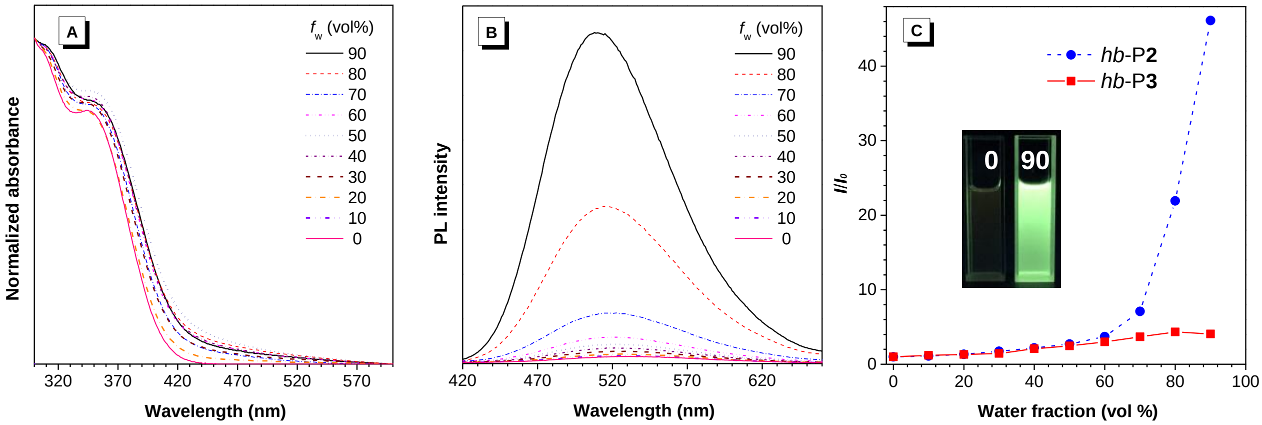

Figure S15. (A) Absorption and (B) Photoluminescence (PL) spectra of *hb*-P**2** in THF and THF/water mixtures. Concentration: ~10 μM. λ_{ex}: 350 nm. (C) Plot of relative PL intensity *versus* water fraction in THF/water mixtures, where I = peak intensity in water mixtures and I_0 = peak intensity in pure THF. Inset: photographs of *hb*-P**2** in pure THF and a THF/water mixture with 90% water fraction.

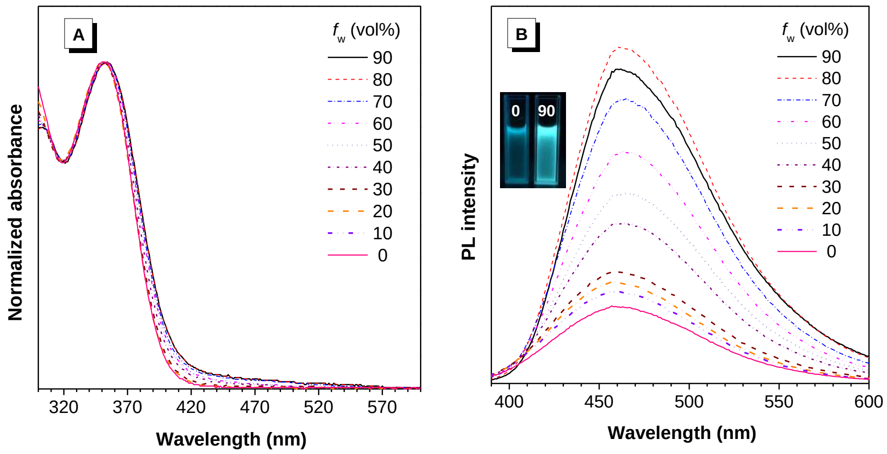

Figure S16. (A) Absorption and (B) PL spectra of *hb*-P**3** in THF/water mixtures with different water fraction. Concentration: ~10 µM. λ_{ex}: 352 nm. Inset: photographs of *hb*-P**3** in pure THF and a THF/water mixture with 90% water fraction.

Table S4. Photophysical properties of *hb*-PAs.

	λ_{ab}(nm)[a]		λ_{em} (nm)[b]		Φ_F[d] (%)	
	THF[c]	film	THF[c]	film	THF[c]	film
hb-P**1**	364	372	428	578	31	5.5
hb-P**2**	350	355	531	506	1.4	49
hb-P**3**	352	361	467	458	2.1	4.1

[a] λ_{ab} = absorption maximum. [b] λ_{em} = emission maximum. [c] In dilute THF solution (10 µM). [d] Absolute fluorescence quantum yield measured by an integrating sphere.

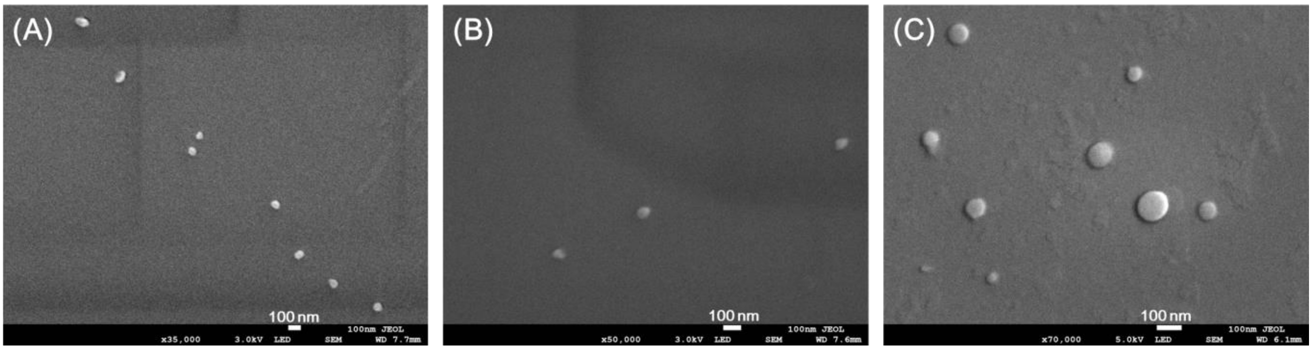

Figure S17. Scaning electron microscope (SEM) images of the aggregates of (A) *hb*-P**1**, (B) *hb*-P**2** and (C) *hb*-P**3**, which were formed in THF/water mixtures with 90% water fraction. Concentration: 10 µM.

Procedures of site-selective three-step functionalization of *hb*-P1

hb-P**1-1**: Into a 10 mL dried Schlenk tube equipped with magnetic stirrer was placed with *hb*-P**1** (60 mg, Table S2, entry 2) and benzylamine (60 mg). Then, dried DMAc (1 mL) was injected into the tube by

pipette. The resultant mixture was stirred at 80 °C in air for 5 h. After cooled to room temperature, 4 mL of DCM was added to dilute the mixture. Then the solution was added dropwise into 200 mL of methanol via a cotton filter under stirring. The precipitate was allowed to stand overnight and then collected by filtration. The polymer was washed with methanol and dried under vacuum at room temperature to a constant weight. A yellow solid was obtained (74.9 mg). M_w: 14 900, M_w/M_n: 1.79 (GPC, polystyrene calibration). ^{1}H NMR (500 MHz, CD_2Cl_2), δ (TMS, ppm):8.85, 7.49-6.91, 4.66, 4.33, 4.03, 3.10, 1.72-1.43, 1.42-1.15.

hb-P**1-2**: Into a 10 mL dried Schlenk tube equipped with magnetic stirrer was placed with *hb*-P**1-1** (73 mg), 1-bromooctane (115.9 mg, 0.6 mmol), Ag_2WO_4 (7.0 mg, 0.015 mmol) and Cs_2CO_3 (195.5 mg, 0.6 mmol) under CO_2 (balloon). Then, dried DMAc (1 mL) was injected into the tube by a syringe. The resultant mixture was stirred at 80 °C under atmospheric CO_2 for 12 h. After cooled to room temperature, 4 mL of DCM was added to dilute the mixture. Then the solution was added dropwise into 200 mL of methanol via a cotton filter under stirring. The precipitate was allowed to stand overnight and then collected by filtration. The polymer was washed with methanol and dried under vacuum at room temperature to a constant weight. A yellow solid was obtained (77.5 mg). M_w: 15 900, M_w/M_n: 2.25 (GPC, polystyrene calibration). ^{1}H NMR (500 MHz, CD_2Cl_2), δ (TMS, ppm):8.86, 7.62-6.70, 4.67, 4.34, 4.18, 4.02, 1.77-1.02, 0.86.

hb-P**1-3**: Into a 10 mL dried Schlenk tube equipped with magnetic stirrer was placed with *hb*-P**1-2** (60 mg) and morpholine (60 mg). Then, dried DMAc (1 mL) was injected into the tube by pipette. The resultant mixture was stirred at 80 °C under air for 12 h. After cooled to room temperature, 4 mL of DCM was added to dilute the mixture. Then the solution was added dropwise into 200 mL of methanol via a cotton filter under stirring. The precipitate was allowed to stand overnight and then collected by filtration. The polymer was washed with methanol and dried under vacuum at room temperature to a constant weight. A yellow solid was obtained (60.7 mg). M_w: 17 400, M_w/M_n: 1.98 (GPC, polystyrene calibration). ^{1}H NMR (500 MHz, CD_2Cl_2), δ (TMS, ppm):8.86, 7.62-6.67, 4.92, 4.67, 4.34, 4.03, 3.86, 3.67, 3.04, 1.74-0.97, .0.86.

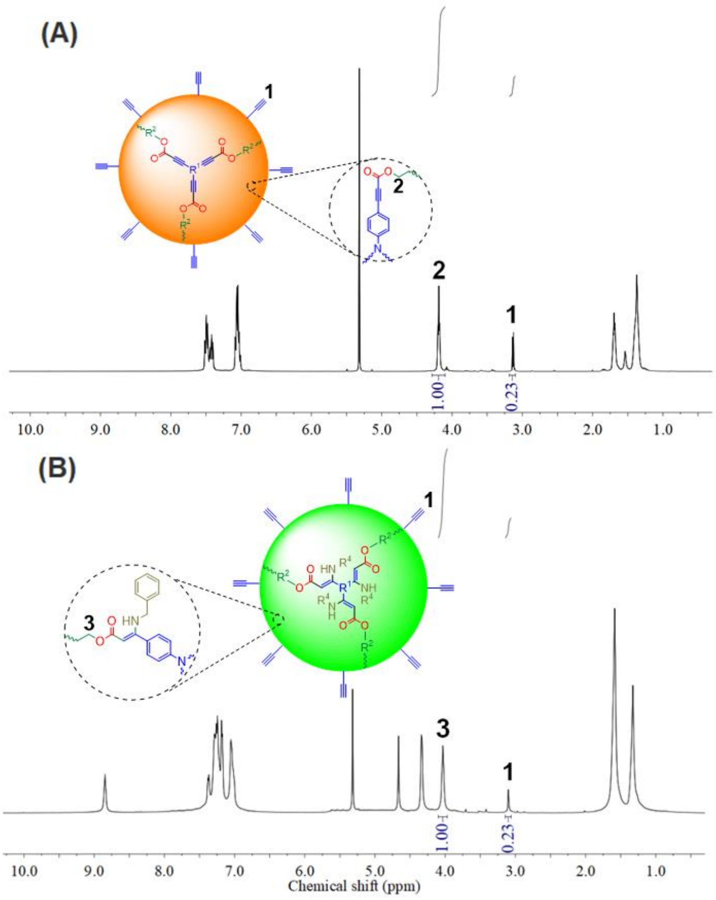

Figure S18. ^{1}H NMR spectra of (A) *hb*-P**1**, (B) *hb*-P**1-1** in DCM-d_2.

Procedures of site-selective tandem polymerization

Into a 10 mL dried Schlenk tube equipped with magnetic stirrer was placed with **1** (63.5 mg, 0.2 mmol), **2** (54.4 mg, 0.2 mmol), Ag_2WO_4 (13.9 mg, 0.03 mmol), Cs_2CO_3 (391.0 mg, 1.2 mmol) under CO_2 (balloon). Dried DMAc (1 mL) was injected into the tube by a syringe. The resultant mixture was stirred at 80 ^oC under atmospheric pressure for 1 h and then followed path **A-C** respectively as shown below.

Path A: Afterward, 1-bromo-2-(2-methoxyethoxy)ethane (73.2 mg, 0.4 mmol) was injected. The mixture was allowed to stir for an additional 12 h at 80 ^oC under CO_2. After cooled to room temperature, 4 mL of DCM was added to dilute the mixture. Then the solution was added dropwise into 200 mL of methanol via a cotton filter under stirring. The precipitate was allowed to stand overnight and then collected by filtration. The polymer was washed with methanol and dried under vacuum at room temperature to a constant weight. A yellow solid *hb*-P**1-4** was obtained (115.6 mg). M_w: 9200, M_w/M_n:

1.49 (GPC, polystyrene calibration). ^{1}H NMR (500 MHz, CD_2Cl_2), δ (TMS, ppm): 7.59-7.42, 7.15-6.99, 4.33, 4.19, 3.71, 3.62, 3.52, 3.35, 1.70, 1.37.

Path B: Afterward, methyl-PEG3-amine (130.6 mg, 0.8 mmol) was injected. The mixture was allowed to stir for an additional 6 h in air. After cooled to room temperature, 4 mL of DCM was added to dilute the mixture. Then the solution was added dropwise into 200 mL of methanol via a cotton filter under stirring. The precipitate was allowed to stand overnight and then collected by filtration. The polymer was washed with methanol and dried under vacuum at room temperature to a constant weight. A yellow solid *hb*-P**1-5** was obtained (148.8 mg). M_w: 7100, M_w/M_n: 1.56 (GPC, polystyrene calibration). ^{1}H NMR (500 MHz, CD_2Cl_2), δ (TMS, ppm): 8.64, 7.46-7.23, 7.20-6.97, 4.60, 4.04, 3.66-3.41, 3.31, 3.13, 1.62, 1.35.

Path C: Afterward, 1-bromo-2-(2-methoxyethoxy)ethane (73.2 mg, 0.4 mmol) was injected. The mixture was allowed to stir for an additional 12 h at 80 ^oC under CO_2. Then methyl-PEG3-amine (195.9 mg, 1.2 mmol) was injected. The mixture was allowed to stir for an additional 6 h in air. After cooled to room temperature, 4 mL of DCM was added to dilute the mixture. Then the solution was added dropwise into 200 mL of methanol via a cotton filter under stirring. The precipitate was allowed to stand overnight and then collected by filtration. The polymer was washed with methanol and dried under vacuum at room temperature to a constant weight. A yellow solid *hb*-P**1-6** was obtained (199.7 mg). M_w: 9800, M_w/M_n: 1.53 (GPC, polystyrene calibration). ^{1}H NMR (500 MHz, CD_2Cl_2), δ (TMS, ppm): 8.64, 7.37-7.24, 7.19-7.02, 4.60, 4.19, 4.04, 3.72-3.39, 3.31, 1.62, 1.34.

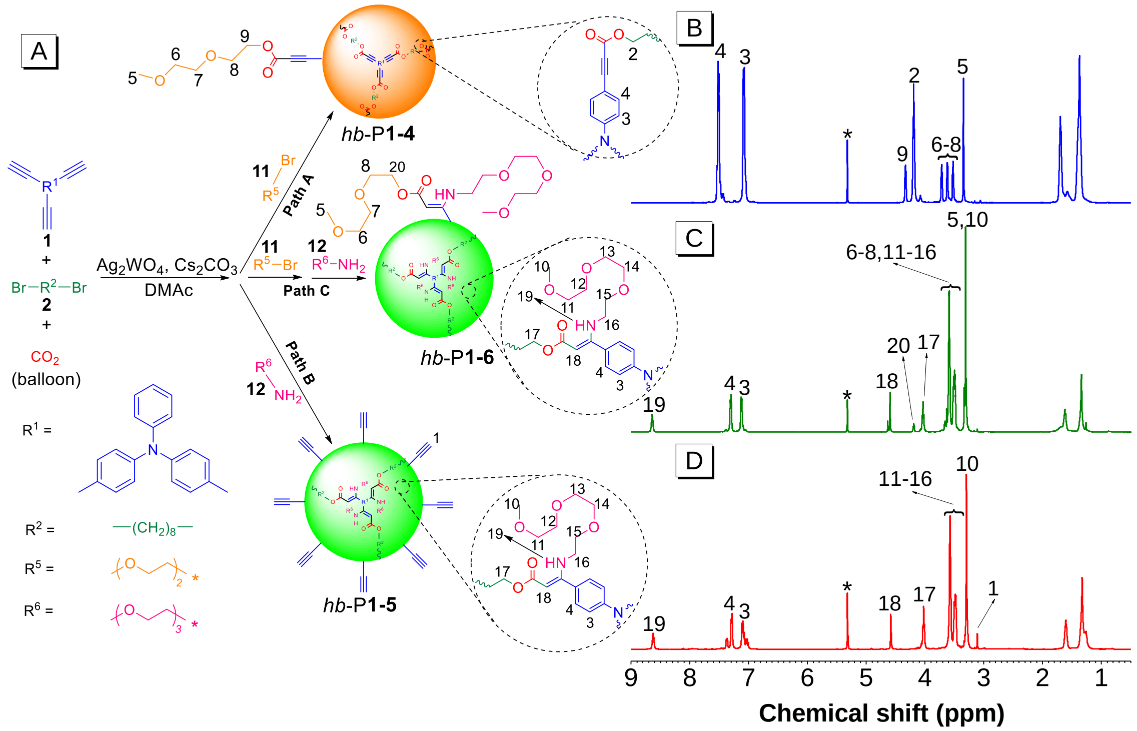

Figure S19. (A) Site-selective tandem polymerizations toward HBPs containing hydrophilic OEG chains in the interior or periphery or both of them and ^{1}H NMR spectra of (B) polymer *hb*-P**1-4**, (C) *hb*-P**1-6** and (D) *hb*-P**1-5** in DCM-d_2. The solvent peaks are marked with asterisks.

Synthesis of hyperbranched polyprodrug amphiphiles

Into a 10 mL dried Schlenk tube equipped with magnetic stirrer were placed with **1** (63.5 mg, 0.2 mmol), **2** (54.4 mg, 0.2 mmol), Ag_2WO_4 (13.9 mg, 0.03 mmol), Cs_2CO_3 (391.0 mg, 1.2 mmol) under CO_2 (balloon). Dried DMAc (1 mL) was injected into the tube by a syringe. The resultant mixture was stirred at 80 ^oC under atmospheric pressure for 30 minutes. Afterward, mPEG-Br (50 mg) was injected. The mixture was allowed to stir for an additional 24 h at 80 ^oC under CO_2. Then 1-butyl iodide (73.6 mg, 0.4 mmol) was injected. The mixture was allowed to stir for an additional 12 h at 80 ^oC under CO_2. After cooled to room temperature, the insoluble mixture was removed by filtration. Then the solution was added dropwise into DI water under vigorous stirring. DMAc and other small molecules were removed by dialysis (MWCO 8000 Da) against DI water for 24 h. A yellow solid *hb*-P**1-7** was obtained after rotary evaporation (125.4 mg). M_w: 18 500, M_w/M_n: 2.46 (GPC, polystyrene calibration). ^{1}H NMR (500 MHz, CD_2Cl_2), δ (TMS, ppm): 7.58-7.33, 7.17-6.91, 4.34, 4.19, 3.60, 3.34, 1.75-1.55, 1.49-1.18, 0.86.

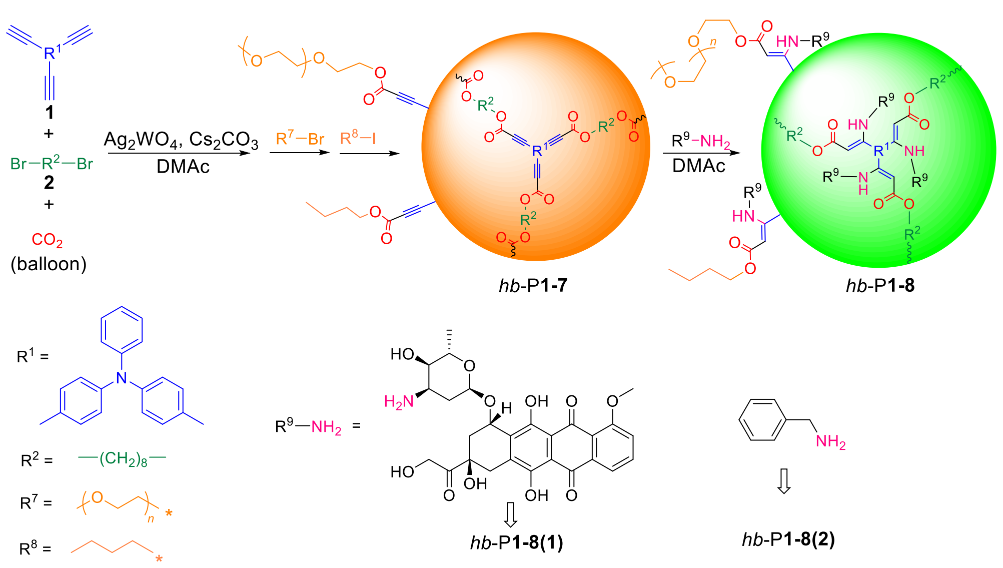

Scheme S4. Synthesis of hyperbranched polyprodrug amphiphiles via site-selective multi-step functionalizations of *hb*-P**1**.

hb-P**1-8(1)**: Into a 10 mL dried Schlenk tube equipped with magnetic stirrer were placed with *hb*-P**1-7** (30 mg) and DOX (60 mg). Then, dried DMAc (1 mL) was injected into the tube by a syringe. The resultant mixture was stirred at 40 °C in air for 2 days. After cooled to room temperature, the solution was added dropwise into DI water under vigorous stirring. DMAc and other small molecules were removed by dialysis (MWCO 3500 Da) against DI water for 24 h. A deep red solid *hb*-P**1-8(1)** was obtained after rotary evaporation (51.7 mg). M_w: 18 700, M_w/M_n: 2.35 (GPC, polystyrene calibration). ^{1}H NMR (500 MHz, DMSO-d_6), δ (TMS, ppm): 8.70, 8.53-6.89, 5.70-5.07, 4.84, 4.65, 4.17, 4.09-3.72, 3.51, 2.95, 2.30-0.51.

hb-P**1-8(2)**: Into a 10 mL dried Schlenk tube equipped with magnetic stirrer were placed with *hb*-P**1-7** (30 mg) and benzylamine (30 mg). Then, dried DMAc (1 mL) was injected into the tube by a syringe. The resultant mixture was stirred at 80 °C in air for 5 h. After cooled to room temperature, the solution was added dropwise into DI water under vigorous stirring. DMAc and other small molecules were removed by dialysis (MWCO 3500 Da) against DI water for 24 h. A yellow solid *hb*-P**1-8(2)** was obtained after rotary evaporation (36.1 mg). M_w: 17 100, M_w/M_n: 2.20 (GPC, polystyrene calibration). ^{1}H NMR (500 MHz, CD_2Cl_2), δ (TMS, ppm): 8.85, 7.47-6.93, 4.66, 4.33, 4.22, 4.04, 3.60, 3.34, 1.72-1.48, 1.46-1.15, 0.86.

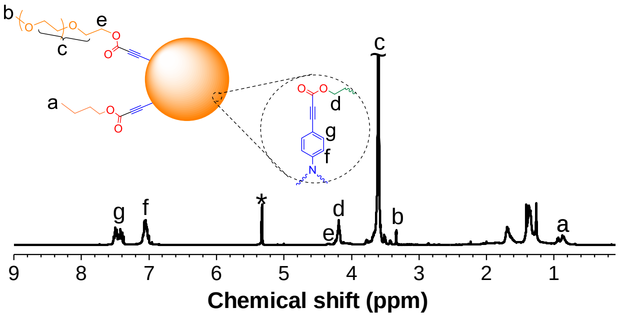

Figure S20. ^{1}H NMR spectrum of polymer *hb*-P**1-7** in DCM-d_2. The solvent peak is marked with an asterisk.

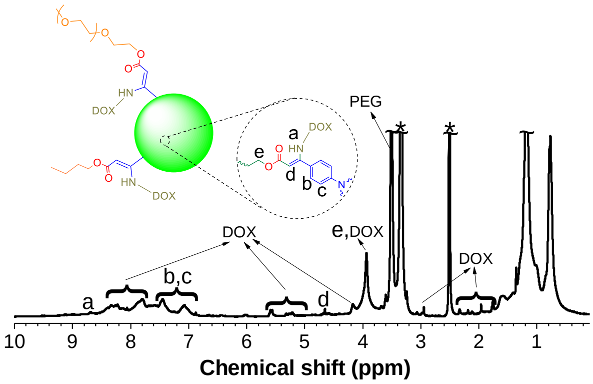

Figure S21. ^{1}H NMR spectrum of polymer *hb*-P**1-8(1)** in DMSO-d_6. The solvent peaks are marked with asterisks.

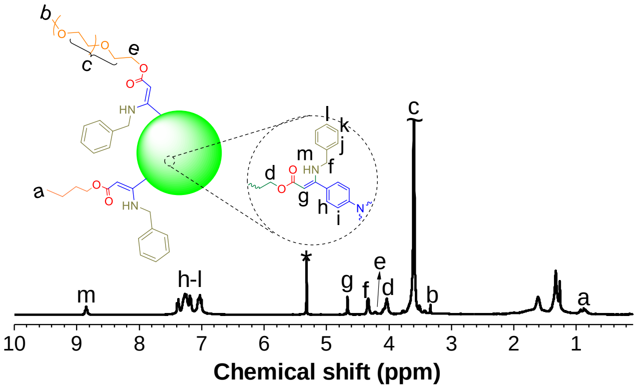

Figure S22. ^{1}H NMR spectrum of polymer *hb*-P**1-8(2)** in DCM-d_2. The solvent peaks are marked with asterisks.

Preparation of hyperbranched polyprodrug NPs

hb-P**1-8(1)** or *hb*-P**1-8(2)** (1 mg) was dissolved in THF (1 mL) and then the solution was added dropwise into DI water (10 mL) under vigorous stirring. The mixed solution was under vigorous stirring for 2 days to make THF volatilize completely. The aggregates were removed by filtration through 0.45-

μm membrane. The hyperbranched polyprodrug NPs were obtained after lyophilization. The DOX content in the NPs were measured for the absorbance at 480 nm by the UV-vis spectrometer. Drug loading content (DLC, wt%) was calculated according to the following formula:

DLC = (amount of loaded drug / amount of drug-loaded NPs) × 100%

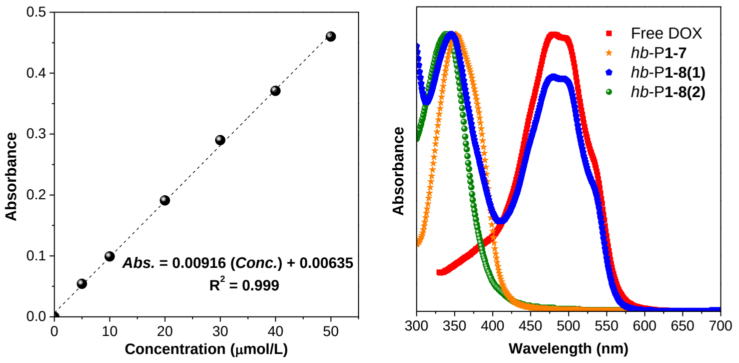

Figure S23. (A) UV-vis calibration curves of free DOX in water and (B) normalized absorption spectra of free DOX, *hb*-P**1-7**, *hb*-P**1-8(1)** and *hb*-P**1-8(2)**. Concentration: ~10 μM.

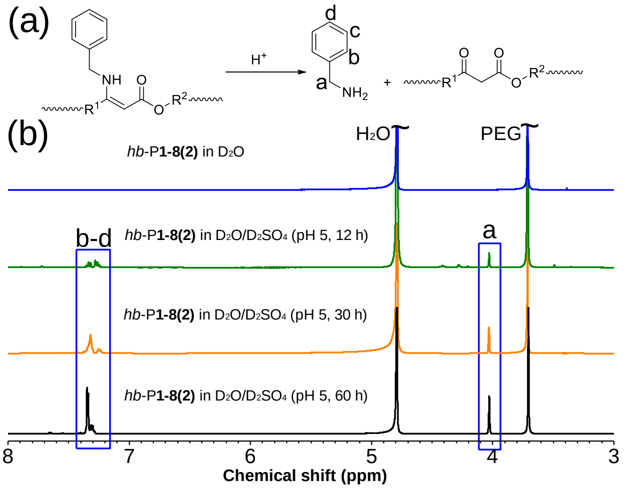

Figure S24. (A) Schematic illustration of acid-triggered removal of amino compound from the polymer and (B) ^{1}H NMR spectra of *hb*-P**1-8(2)** NPs at different time intervals in D_2O/D_2SO_4 with pH 5.0.

In vitro drug release

The drug release from NPs was studied using PBS (pH 7.4 and 5.0). Briefly, *hb*-P**1-8(1)** NPs (5 mL, containing 300 μg DOX) were placed in a dialysis bag (MWCO 3500 Da), which was immersed in 45 mL of the release medium. The release study was performed at 37 ℃ under gentle shaking (100 rpm). At pre-determined time intervals, 4 mL of the release medium was withdrawn and replaced with equal

amount of fresh release medium. The DOX amount in the release medium was determined by UV-vis spectrophotometry.

CLSM study

HeLa cells were incubated with *hb*-P**1-8(1)** or *hb*-P**1-8(2)** NPs (5 μg/mL) in DMEM for 3 h, followed with the incubation of Lysotracker Green DND-26 (200 nM) or Lysotracker Red DND-99 (200 nM) and Hoechst 33342 (1 μM) in DMEM for 30 min, respectively. Then, the spices were observed on CLSM. *hb*-P**1-8(1)** NPs: λ_{ex}: 514 nm, λ_{em}: 580–700 nm. *hb*-P**1-8(2)** NPs: λ_{ex}: 405 nm, λ_{em}: 450–530 nm. Lysotracker Green DND-26: λ_{ex}: 488 nm, λ_{em}: 493–532 nm. Lysotracker Red DND-99: λ_{ex}: 543 nm, λ_{em}: 580–690 nm. Hoechst 33342: λ_{ex}: 405 nm, λ_{em}: 410-480 nm. Overlap coefficient and Pearson correlation coefficient were analyzed on Zeiss LSM 710.

***In vitro* anti-cancer efficacy**

The *in vitro* cytotoxicity of *hb*-P**1-8(1)** or *hb*-P**1-8(2)** NPs was evaluated by the MTT assay. Briefly, HeLa cells were seeded in 96-well plates at 7 000 cells/well and incubated at 37 ℃ for 24 h. The cell culture medium was then replaced with fresh DMEM (200 μL) containing the polymer at the different concentrations. After 48 h incubation, the cell viability was measured by the MTT assay. Cells that did not receive treatment with polymers served as the control, and results were denoted as percentage viability of control cells.

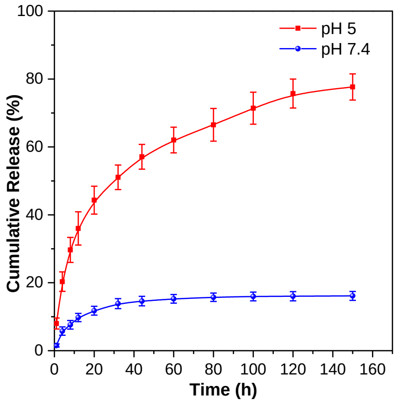

Figure S25. *In vitro* DOX release profiles of *hb*-P**1-8(1)** NPs (n = 3).

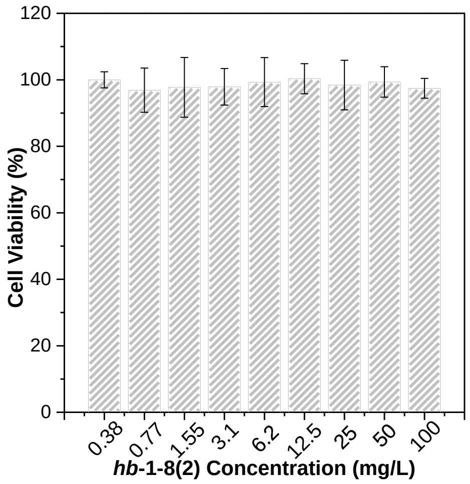

Figure S26. Cytotoxicity of *hb*-P**1-8(2)** NPs toward HeLa cells after incubation for 48 h (n = 5).

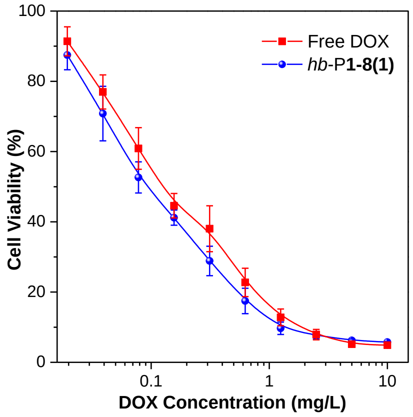

Figure S27. Cytotoxicity of *hb*-P**1-8(1)** NPs and free DOX toward HeLa cells after incubation for 48 h (n = 5).

Statistical analysis

Experiments were repeated for at least three times and results were expressed as means ± SD. Statistical significances were analyzed using the Student's *t*-test, and differences between the test and control groups were judged to be significant at $*p < 0.05$. The Student's *t*-test was carried out using Origin9 Software.

Construction of artificial light-harvesting system

Into 10 mL dried Schlenk tubes equipped with magnetic stirrer were placed with **1** (63.5 mg, 0.2 mmol), **2** (54.4 mg, 0.2 mmol), Ag_2WO_4 (13.9 mg, 0.03 mmol), Cs_2CO_3 (391.0 mg, 1.2 mmol) under CO_2 (balloon). Dried DMAc (1 mL) was injected into the tubes by a syringe. The resultant mixtures were stirred at 80 ^{o}C under atmospheric pressure for 1 h. Afterward, different amounts of C343-Br was added,

respectively. The mixtures were allowed to stir for an additional 12 h at 100 °C under CO_2. After cooled to room temperature, 4 mL of DCM was added to dilute the mixture. Then the solution was added dropwise into 200 mL of methanol via a cotton filter under stirring. The precipitate was allowed to stand overnight and then collected by filtration. The polymer was washed with methanol and dried under vacuum at room temperature to a constant weight. Yellow solids *hb*-PA-(x_{C343}/y_{TPA}) was obtained.

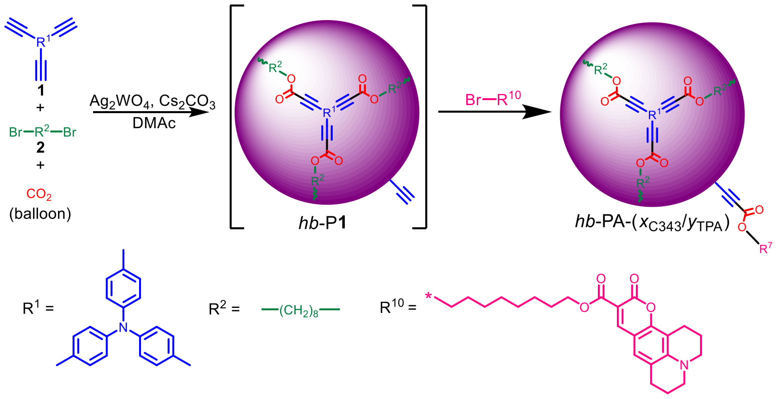

Scheme S5. Construction of artificial light-harvesting system via a "one-pot" tandem reaction strategy.

Preparation of fluorophores C343-Br

The synthetic routes to C343-Br are show in Scheme S6. Into a 250 mL two-necked round bottom flask equipped a condenser, 1,8-dibromooctane (163.2 mg, 0.6 mmol), potassium carbonate (55.3 mg, 0.4 mmol) and 20 mL acetone were added. Then reaction mixture was refluxed and C343 (57.1 mg, 0.2 mmol) dissolved in 40 mL acetone was added via a dropping funnel under stirring. After finishing the addition, the mixture was stirred for additional 6 h and then cooled to room temperature. After filtration and solvent evaporation, the crude product was purified by a silica gel column chromatography using petroleum ether/ethyl acetate (1:1 v/v) as eluent. A yellow solid C343-Br was obtained in 42% yield (40 mg). ^{1}H NMR (500 MHz, CD_2Cl_2), δ (TMS, ppm): 8.29 (s, 1H), 6.96 (s, 1H), 4.23 (t, J = 6.7 Hz, 2H), 3.42 (t, J = 6.9 Hz, 2H), 3.37 – 3.25 (m, 4H), 2.84 (t, J = 6.4 Hz, 2H), 2.75 (t, J = 6.2 Hz, 2H), 1.96 (m, 5.9 Hz, 4H), 1.90 – 1.80 (m, 2H), 1.79 – 1.67 (m, 2H), 1.48 – 1.36 (m, 8H). ^{13}C NMR (125 MHz, CD_2Cl_2), δ (TMS, ppm): 164.60, 158.65, 153.84, 149.27, 148.92, 127.28, 119.79, 107.78, 107.71, 106.00, 65.19, 50.67, 50.25, 34.65, 33.28, 30.10, 29.50, 29.05, 28.50, 27.80, 26.31, 21.57, 20.61, 20.42. MS (MALDI-TOF): m/z 475.2 (M^+, calcd 475.1).

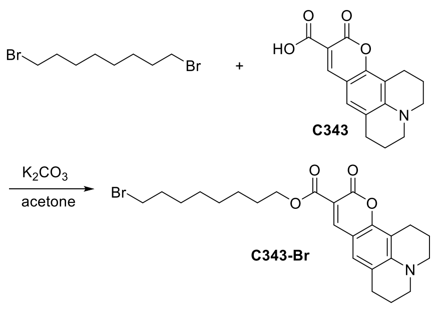

Scheme S6 Synthetic route to C343-Br.

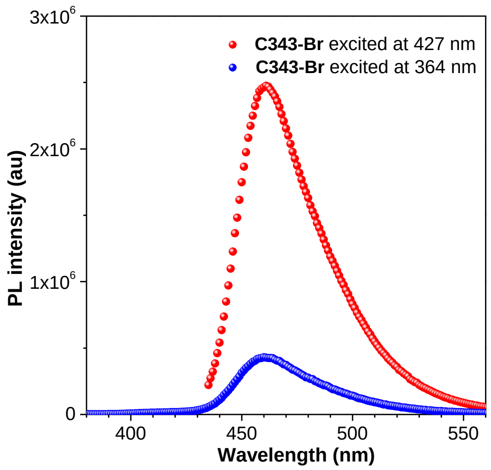

Figure S28. The fluorescence emission spectrum of C343-Br excited at 364 and 427 nm in THF. Concentration: 10 μM.

The energy transfer efficiency was calculated via following equation:

$$\Phi_{\mathrm{ET}} = \left(1 - \frac{I}{I_0}\right) \times 100\% \qquad \text{(S10)}$$

where Φ_{ET} is the energy transfer efficiency. Keeping TPA molar concentration of various *hb*-PA-($x_{\mathrm{C343}}/y_{\mathrm{TPA}}$) same, I_0 is the PL intensity of *hb*-PA-($x_{\mathrm{C343}}/y_{\mathrm{TPA}}$=0) at 428 nm, I is the PL intensity of other *hb*-PA-($x_{\mathrm{C343}}/y_{\mathrm{TPA}}$) at 428 nm. In Figure 7C, I_0 = 568450 au, I for *hb*-PA-($x_{\mathrm{C343}}/y_{\mathrm{TPA}}$=0.1), *hb*-PA-($x_{\mathrm{C343}}/y_{\mathrm{TPA}}$=0.2), *hb*-PA-($x_{\mathrm{C343}}/y_{\mathrm{TPA}}$=0.3), *hb*-PA-($x_{\mathrm{C343}}/y_{\mathrm{TPA}}$=0.4) and *hb*-PA-($x_{\mathrm{C343}}/y_{\mathrm{TPA}}$=0.5) are 202180, 184740, 142700, 83460 and 44580 au, respectively. Thus, the Φ_{ET} of *hb*-PA-($x_{\mathrm{C343}}/y_{\mathrm{TPA}}$=0.1), *hb*-PA-($x_{\mathrm{C343}}/y_{\mathrm{TPA}}$=0.2), *hb*-PA-($x_{\mathrm{C343}}/y_{\mathrm{TPA}}$=0.3), *hb*-PA-($x_{\mathrm{C343}}/y_{\mathrm{TPA}}$=0.4) and *hb*-PA-($x_{\mathrm{C343}}/y_{\mathrm{TPA}}$=0.5) are 65%, 68%, 75%, 85% and 92%, respectively.

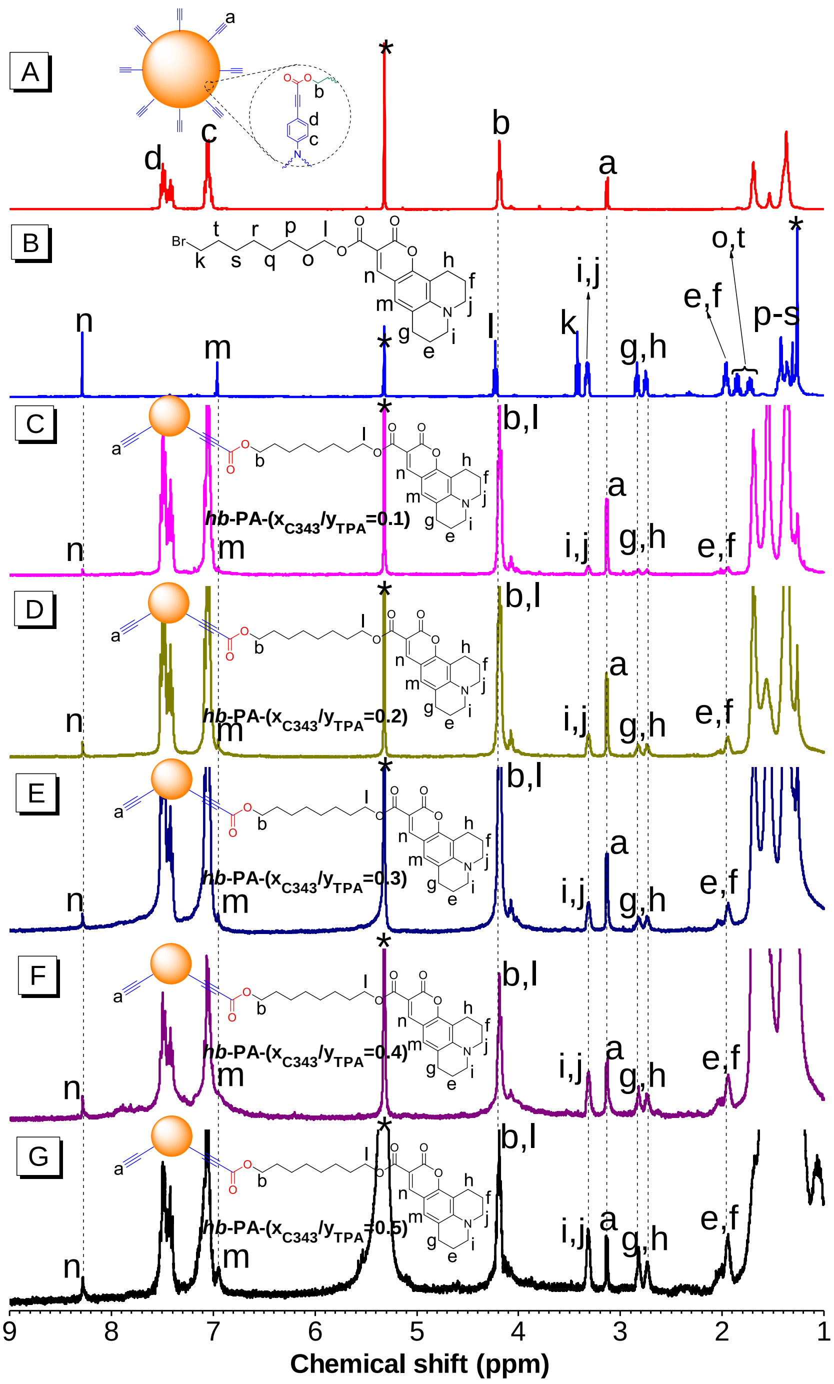

Figure S29. ^{1}H NMR spectra of (A) *hb*-P**1**, (B) C343-Br, (C) *hb*-PA-(x_{C343}/y_{TPA}=0.1), (D) *hb*-PA-(x_{C343}/y_{TPA}=0.2), (E) *hb*-PA-(x_{C343}/y_{TPA}=0.3), (F) *hb*-PA-(x_{C343}/y_{TPA}=0.4) and (G) *hb*-PA-(x_{C343}/y_{TPA}=0.5) in DCM-d_2. The solvent peaks are marked with asterisks.

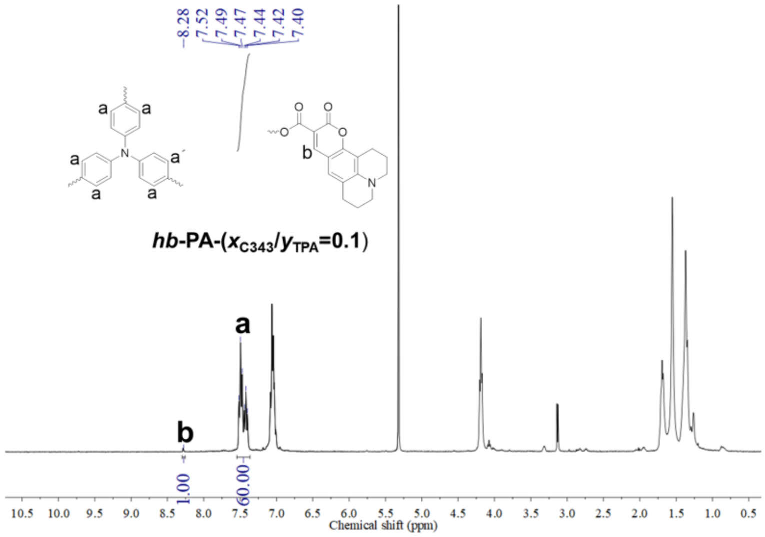

Figure S30. ^{1}H NMR spectrum of *hb*-PA-(x_{C343}/y_{TPA}=0.1) in DCM-d_2.

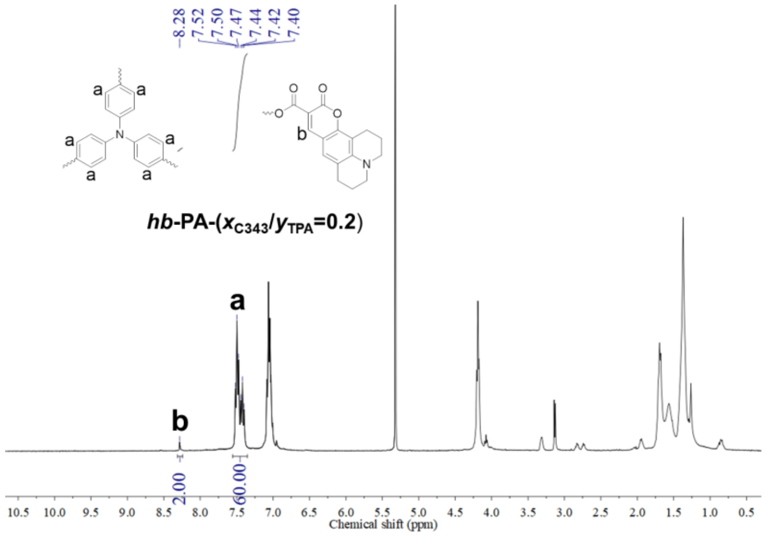

Figure S31. ^{1}H NMR spectrum of *hb*-PA-(x_{C343}/y_{TPA}=0.2) in DCM-d_2.

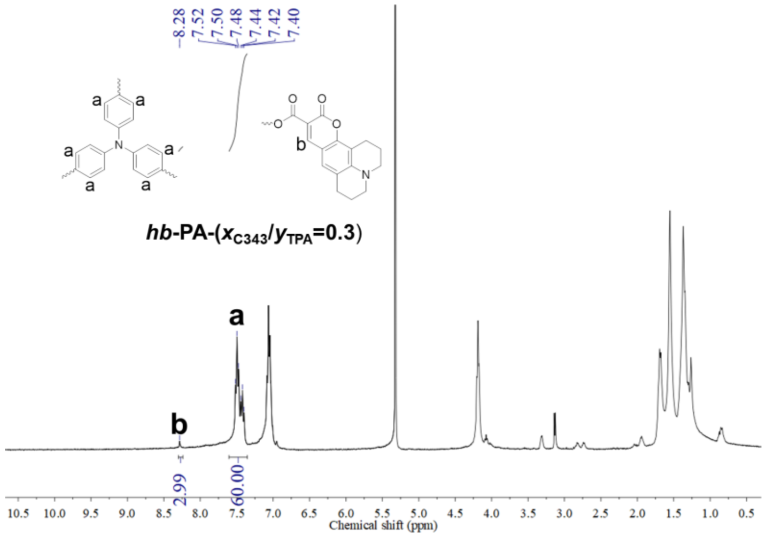

Figure S32. ^{1}H NMR spectrum of *hb*-PA-(x_{C343}/y_{TPA}=0.3) in DCM-d_2.

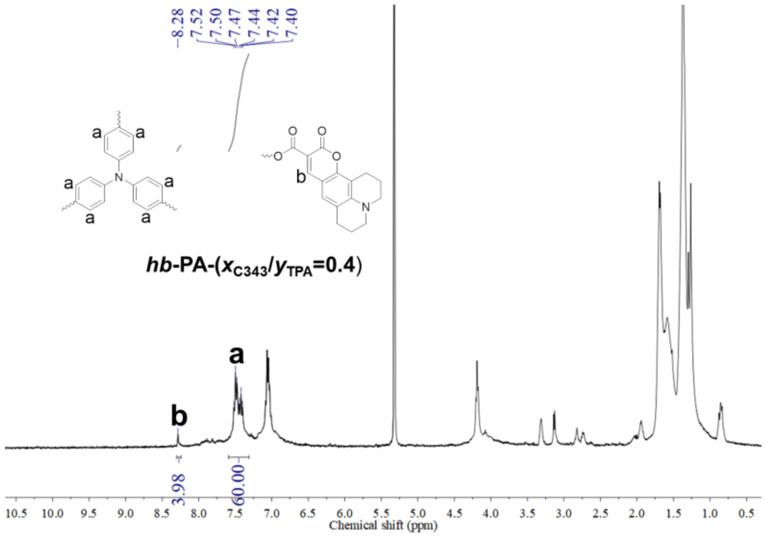

Figure S33. ^{1}H NMR spectrum of *hb*-PA-(x_{C343}/y_{TPA}=0.4) in DCM-d_2.

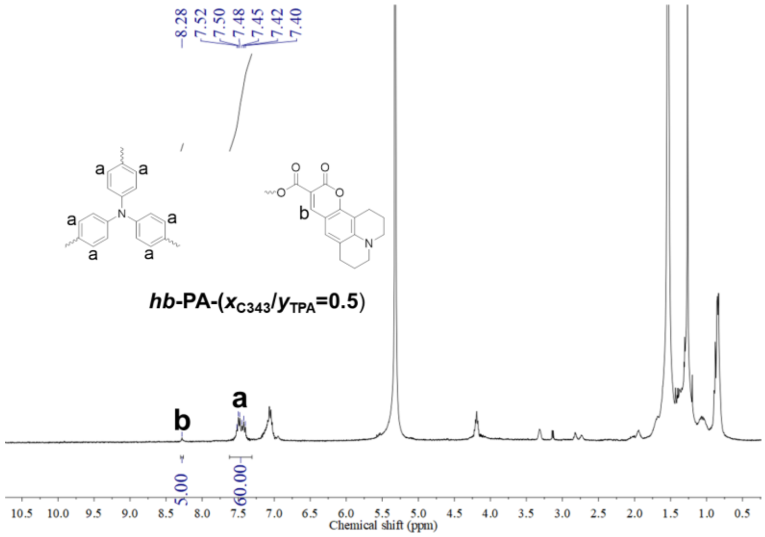

Figure S34. ^{1}H NMR spectrum of *hb*-PA-(x_{C343}/y_{TPA}=0.5) in DCM-d_2.

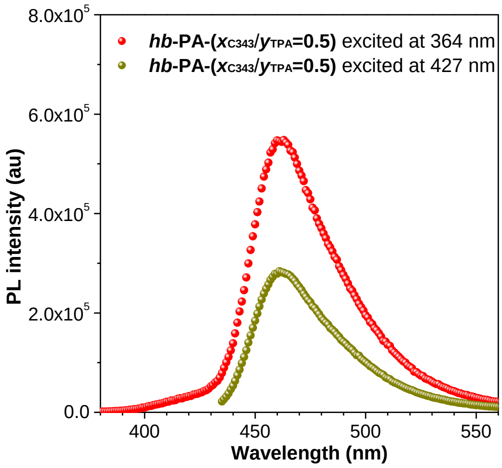

Figure S35. The fluorescence emission spectrum of *hb*-PA-(x_{C343}/y_{TPA}=0.5) excited at 364 and 427 nm in THF. Concentration: ~1 μM.

Preparation of fluorophores 13 and 14

The synthetic routes to **13** and **14** are show in Scheme S7.

1-((8-Bromooctyl)oxy)pyrene (13): Into a 250 mL two-necked round bottom flask equipped a condenser, 1,8-dibromooctane (2.448 g, 9 mmol), potassium carbonate (829.3 mg, 6 mmol) and 80 mL acetone were added. Then reaction mixture was refluxed and 1-hydroxypyreneand (654.8 mg, 3 mmol) dissolved in 40 mL acetone was added via a dropping funnel under stirring. After finishing the addition, the mixture was stirred for additional 8 h and then cooled to room temperature. After filtration and solvent evaporation, the crude product was purified by a silica gel column chromatography using petroleum ether/DCM (3:1 v/v) as eluent. A white solid **13** was obtained in 83% yield (1.019 g). ^{1}H NMR (500 MHz, CD_2Cl_2), δ (TMS, ppm): 8.47 (d, J = 9.1 Hz, 1H), 8.22-7.83 (m, 7H), 7.57 (d, J = 8.4 Hz, 1H), 4.33 (s, 2H), 3.43 (t, J = 6.9 Hz, 2H), 2.07-1.96 (m, 2H), 1.88 (m, 2H), 1.70-1.59 (m, 2H), 1.52-1.36 (m, 6H). ^{13}C NMR (125 MHz, CD_2Cl_2), δ (TMS, ppm): 153.72, 132.13, 127.66, 127.07-124.05, 121.66, 120.67, 109.63, 69.40, 34.66, 33.30, 29.86, 29.68, 29.15, 28.54, 26.59. HRMS (MALDI-TOF): m/z 408.1043 (M^+, calcd 408.1089).

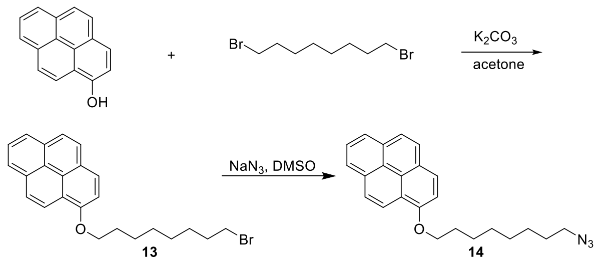

Scheme S7. Synthetic routes to **13** and **14**.

1-((8-Azidooctyl)oxy)pyrene (14): Into a 100 mL round bottom flask were added **13** (409.4 mg, 1 mmol) and sodium azide (97.5 mg, 1.5 mmol) in 50 mL of DMSO. After stirring at room temperature for 12 h, a small amount of water was added to quench the reaction (temperature of the solution increased slightly). After cooled to room temperature, the solution was extracted with 30 mL of diethyl ether five times. The organic phases were combined, washed with water and brine, and then dried over $MgSO_4$ overnight. After filtration and solvent evaporation, the crude product was purified by a silica gel column using petroleum ether/DCM (3:1 v/v) as eluent. A white solid **14** was obtained in 89% yield

(331.7 mg). ^{1}H NMR (500 MHz, CD_2Cl_2), δ (TMS, ppm): 8.49-8.43 (m, 1H), 8.15-7.85 (m, 7H), 7.57 (d, J = 6.7 Hz, 1H), 4.33 (t, J = 5.1 Hz, 2H), 3.26 (t, J = 5.5 Hz, 2H), 2.05-1.97 (m, 2H), 1.70-1.55 (m, 6H), 1.44-1.37 (m, 4H). ^{13}C NMR (125 MHz, CD_2Cl_2), δ (TMS, ppm): 153.73, 132.18, 132.11, 127.66, 126.57, 126.54, 126.13, 126.01, 125.44, 125.30, 125.17, 124.57, 124.45, 121.65, 120.67, 109.63, 69.40, 51.94, 29.87, 29.72, 29.54, 29.23, 27.09, 26.59. HRMS (MALDI-TOF): m/z 371.1969 (M^+, calcd 371.1998).

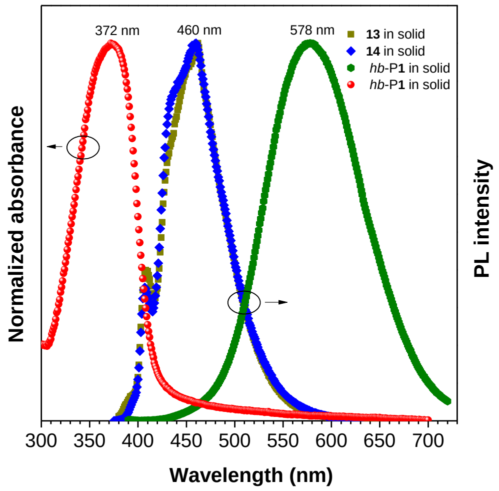

Figure S36. Absorption spectrum of *hb*-P**1** and PL spectrum of *hb*-P**1**, **13** and **14** in their solid states.

Construction of white light-emitting system

hb-P**1-9**: Into a 10 mL dried Schlenk tube equipped with magnetic stirrer were placed with **1** (63.5 mg, 0.2 mmol), **2** (54.4 mg, 0.2 mmol), Ag_2WO_4 (13.9 mg, 0.03 mmol) and Cs_2CO_3 (391.0 mg, 1.2 mmol) under CO_2 (balloon). Dried DMAc (1 mL) was injected into the tube by a syringe. The resultant mixture was stirred at 80 ^{o}C under atmospheric pressure for 1 h. Afterward, **13** (245.6 mg, 0.6 mmol) was added. The mixture was allowed to stir for an additional 12 h at 100 ^{o}C under CO_2. After cooled to room temperature, 4 mL of DCM was added to dilute the mixture. Then the solution was added dropwise into 200 mL of methanol via a cotton filter under stirring. The precipitate was allowed to stand overnight and then collected by filtration. The polymer was washed with methanol and dried under vacuum at room temperature to a constant weight. A yellow solid was obtained (144.3 mg). M_w: 16 700, M_w/M_n: 1.83 (GPC, polystyrene calibration). ^{1}H NMR (500 MHz, CD_2Cl_2), δ (TMS, ppm): 8.50-8.43, 8.16-7.85, 7.62-7.37, 7.13-6.98, 4.33, 4.20, 4.10, 2.08-1.82, 1.77-1.29.

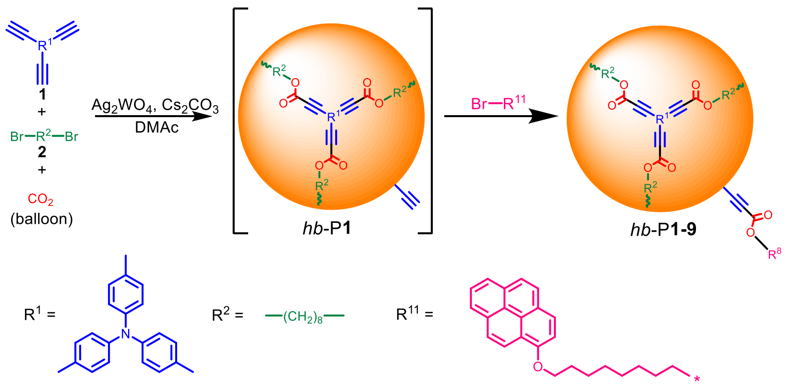

Scheme S8. Construction of white light-emitting system via a “one-pot” tandem reaction strategy.

hb-P**1-10**: Into a 10 mL dried Schlenk tube equipped with magnetic stirrer were placed with *hb*-P**1** (51.5 mg), **14** (111.5 mg, 0.3 mmol) and $Cu(PPh_3)_3Br$ (9.3 mg, 0.01 mmol) under nitrogen. Then, dried DMF (1 mL) was injected into the tube by a syringe. The resultant mixture was stirred at 60 ^oC under nitrogen for 12 h. After cooled to room temperature, 4 mL of DCM was added to dilute the mixture. Then the solution was added dropwise into 200 mL of methanol via a cotton filter under stirring. The precipitate was allowed to stand overnight and then collected by filtration. The polymer was washed with methanol and dried under vacuum at room temperature to a constant weight. A yellow solid was obtained (82.2 mg). M_w: 17 400, M_w/M_n: 1.86. ^1H NMR (500 MHz, CD_2Cl_2), δ (TMS, ppm): 8.52-8.38, 8.18-7.36, 7.22-6.93, 4.43-4.23, 4.18, 2.08-1.79, 1.78-1.14.

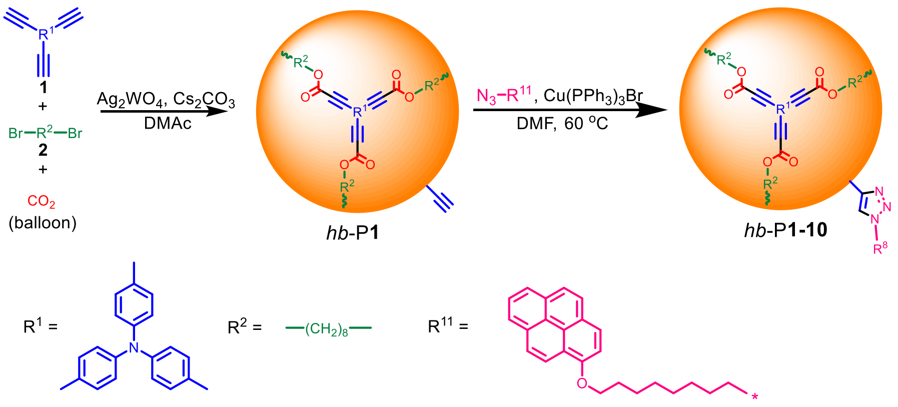

Scheme S9. Construction of white light-emitting system via a “step-by-step” reaction strategy.

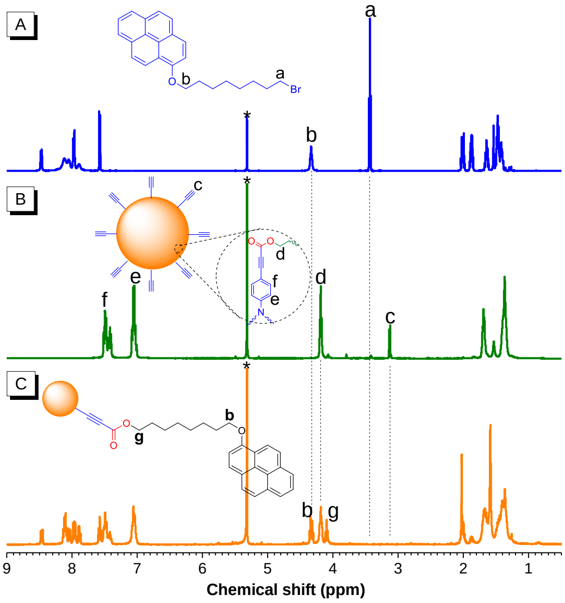

Figure S37. ^{1}H NMR spectra of (A) **13**, (B) *hb*-P**1** and (C) *hb*-P**1-9** in DCM-d_2. The solvent peaks are marked with asterisks.

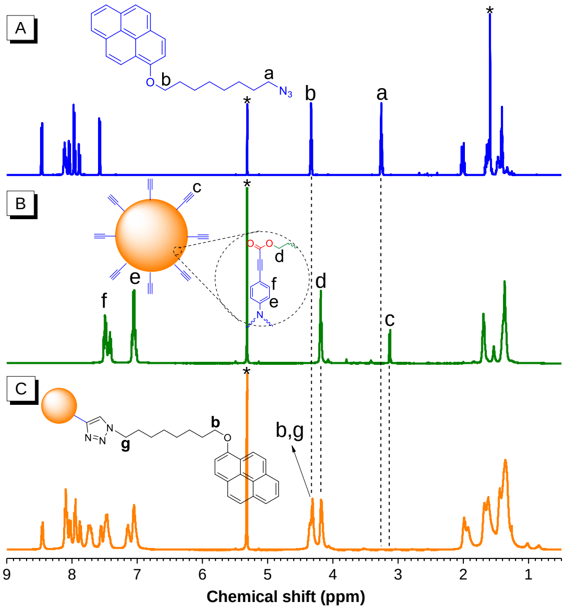

Figure S38. ^{1}H NMR spectra of (A) **14**, (B) *hb*-P**1** and (C) *hb*-P**1-10** in DCM-d_2. The solvent peaks are marked with asterisks.

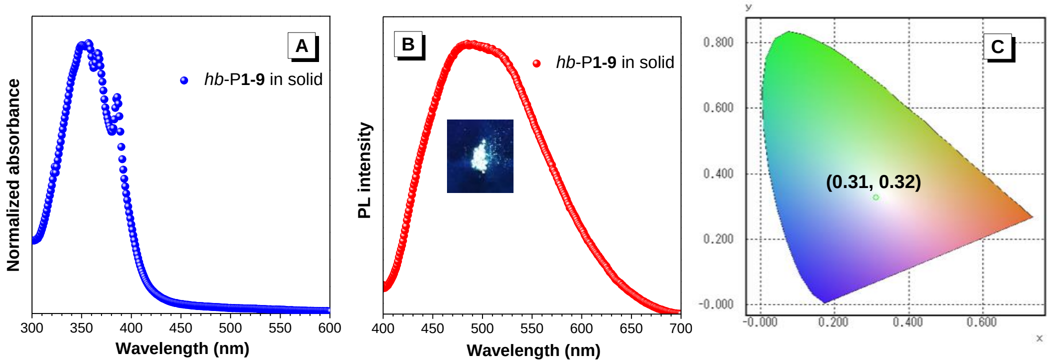

Figure S39. (A) Absorption and (B) PL spectra of *hb*-P**1-9** in solid states. λ_{ex}: 365 nm. (C) CIE 1931 coordinates in CIE-1931 chromaticity diagram of white emissive *hb*-P**1-9**.

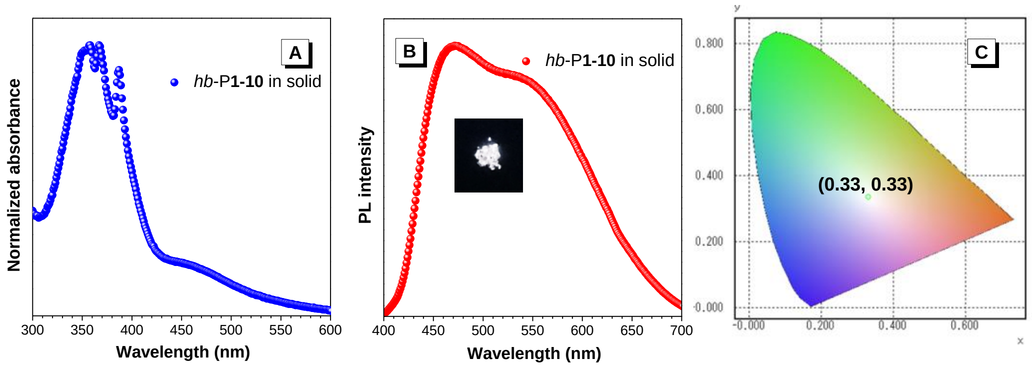

Figure S40. (A) Absorption and (B) PL spectra of *hb*-P**1-10** in solid states. λ_{ex}: 365 nm. (C) CIE 1931 coordinates in CIE-1931 chromaticity diagram of white emissive *hb*-P**1-10**.

References

[1] B. Yao, T. Hu, H. Zhang, J. Li, J. Z. Sun, A. Qin, B. Z. Tang, *Macromolecules* **2015**, *48*, 7782-7791.
